# Microstructure modeling and computational micromechanics of glued metallic hollow-sphere composites

**DOI:** 10.1007/s00419-026-03139-9

**Published:** 2026-06-29

**Authors:** Lukas Jabs, Xin Zhou, Lars Penter, Steffen Ihlenfeldt, Matti Schneider

**Affiliations:** 1 Institute of Engineering Mathematics, University of Duisburg-Essen, Duisburg, Germany; 2 Institute of Mechatronic Engineering, TU Dresden, Dresden, Germany; 3 Fraunhofer Institute for Machine Tools and Forming Technology IWU, Chemnitz, Germany; 4 Center for Nanointegration Duisburg-Essen (CENIDE), Duisburg, Germany; 5 Fraunhofer Institute for Industrial Mathematics ITWM, Kaiserslautern, Germany

**Keywords:** Hollow-sphere composites, Porous material, FFT-based computational micromechanics, Level-set method, Particle-filled hollow spheres

## Abstract

This work is devoted to generating and investigating microstructure representations of glued hollow-sphere assemblies, which combine low weight with excellent damping properties for use in machine tools. As a first step, we model the microstructure of such assemblies based on a dedicated analysis of the size distribution of the hollow spheres. To model the glue, we augment a preliminary sphere packing with dedicated closing and erosion operations, prevalent in mathematical morphology. Notably, for spheres, a level-set representation of the result of the closing and erosion operations can be computed analytically based on specific Dirichlet diagrams. In this way, we obtain a resolution-independent description of the glue phase for irregular sphere arrangements. Once the microstructure is generated, we furnish the individual phases with the elastic properties of the involved materials. We use FFT-based computational micromechanics tools to compute the effective elastic properties of the assemblies and study the influence of various factors, like the sphere-size distribution, the thickness of the shell, and the employed material parameters. Of particular importance here is the use of composite voxels, which in turn rely on the previously derived level-set representation. The developed modeling approach determines the elastic material properties of the hollow-sphere composite (HSC) and identifies the leading influences as the shell thickness, the glue phase volume fraction, and the sphere packing density. This approach aligns well with the validated structure, which has an average sphere diameter of 2.85 mm, a wall thickness of 72 $\upmu$m, a sphere packing density of 54%, and an epoxy volume fraction of 16.2%.

## Introduction

### State of the art

Production efficiency in modern machine tools is commonly enhanced through higher feed rates, which lead to significant increases in acceleration fluctuations and structural vibrations, thereby reducing the machining accuracy. To maintain the necessary precise geometric configuration while absorbing vibrational disturbances, therefore, requires stiff and highly damping materials [1]. Moving machine parts should also be lightweight to allow for high accelerations, e.g., high cornering speeds [2, 3]. Among several proposed damping strategies [4–7], sandwich composites with a core of metal foams or cellular materials are particularly promising [8]. The face sheets provide stiffness while the core offers favorable damping behavior [9], and the resulting weight-specific stiffness exceeds that of traditional materials such as steel, gray cast iron, and mineral cast iron by more than an order of magnitude [1].

A special class of cellular materials are hollow sphere composites, where – as the name suggests – hollow spheres are joined together to form a composite. The spheres are manufactured by chemical or electrical deposition [10], or by coating a placeholder with a metal [11] or ceramic [12] powder-binder mixture, followed by sintering [13], and they are joined by epoxy [14, 15], sintering [16], or brazing [17]. The resulting structures form lightweight, foam-like cores with high damping behavior [18]. A comprehensive review for metallic HSCs is available [19]. Experimental characterizations have addressed yield strength, microporosity, alternative material systems, coatings, and densification[11, 13, 16, 20–22]. Filling the metallic spheres with mineral or ceramic particles yields particle-filled hollow sphere structures (PHSS) [23], in which additional energy dissipates through particle-particle and particle-shell collisions, further enhancing damping[15, 23, 24].

Complementing the experimental investigations, a number of theoretical and numerical investigations were conducted to determine the mechanical properties of HSCs. For instance, exploiting formal similarities of HSCs and foams, mechanical properties were estimated by heuristic formulas developed for foams, see the works [12, 23, 25] and Gibson-Ashby [26, §5]. Several studies utilizing finite element method (FEM) models were conducted on regularly arranged HSCs and cellular structures to determine the yield strength [27–30], the creep  [31], and the buckling behavior [32], the thermal conductivity [14], and the fracture behavior [20]. Furthermore, the compression of a single hollow sphere [33] and the impact behavior of an HSC [34] were investigated. To determine the acoustic properties, tube absorption methods were used [35].

To determine the mechanical properties of a material accurately, the proper microstructure should be taken into account [36]. The aforementioned contributions all restrict to regular sphere arrangements, presumably due to restrictions on the computational feasibility. Key features of industrial PHSS include random filler size distribution, imperfections and manufacturing-induced variations. Simplified microstructural models neglect these aspects and, as a result, fail to accurately represent real microstructures or to capture their influence on effective material properties.

Different approaches were developed to model random microstructures, in particular accounting for non-uniform sphere sizes and irregular arrangements. The simplest method is random sequential adsorption (RSA) [37], which places spheres one by one such that no overlap with previously placed spheres occurs, terminating once the target volume fraction is reached. RSA cannot reach high packing densities, which motivated dynamic and contraction-based alternatives [38–40]. The sequential addition and migration method (SAM) [41, 42], originally developed for fiber-reinforced composites and equally applicable to spheres, achieves significantly higher packing densities than RSA by adding only a portion of the inclusions at a time and removing overlaps before the next addition step.

In addition to the spheres of the HSCs, the glued adhering them must be modeled as well. In previous contributions [43, 44], an organic interface between spheres is modeled via a thin, uniform outer layer for regular and random sphere arrangements. To account for the connectivity of pores, one may use operators from mathematical morphology [45], specifically the closing operation [46, §4]. To represent the binder in casting applications, the closing was performed on a digitized microstructure [47] or an approximation of the level-set function was utilized [48]. Morphological operations on spheres result in overlapping sphere assemblies. For these assemblies, analytical analyses for advantageous decompositions and inclusion-exclusion strategies exist [49, 50]. These strategies were originally proposed for the description of molecules [51].

In summary, HSCs demonstrate excellent lightweight properties, and particle filling enhances their damping capabilities. Tailored experiments provided numerous insights into the material properties. Such experiments may be complemented by dedicated computational approaches, in particular to cover a wider range of variations within the material at a comparatively low cost. However, existing approaches do not provide microstructure representations that simultaneously capture

(i) irregular sphere arrangements,
(ii) thin interphases between particles, and
(iii) efficient evaluation within computational homogenization frameworks.

These shortcomings limit their applicability for realistic HSCs.

### Contributions

To overcome these limitations, we develop a microstructure modeling framework for glued hollow-sphere assemblies that combines an analytic level-set representation with an FFT-based homogenization approach.

The computational studies for HSCs presented in the introductory section 1.1 offer valuable insights; however, they only consider highly idealized microstructures, such as grid-like sphere arrangements or systems with only small epoxy fractions. In particular, the typically considered cubic sphere arrangement with isotropic constituent materials leads to an effective stiffness tensor which reflects the cubic symmetry of the microstructure, i.e., the proposed microstructure model does not match the isotropic material behavior observed for real HSCs [1]. Therefore, we aim to create random sphere assemblies with no preferred direction(s) and analyze their apparent elastic material properties. Both Schneider et al. [47] and Donval et al. [48] consider irregular geometric arrangements of sand grains that are bonded together by an inorganic binder and modeled using a morphological operator. We take a similar approach, determining the binder/epoxy phase via a closing operation with an exact level-set function in the continuous setting. We introduce a projection scheme to determine the level-set function for a union of overlapping balls. This microstructure representation is then used to predict the macroscopic behavior of hollow sphere composites based on their microstructure, specifically, the linear elastic properties of randomly arranged hollow spheres (HS) joined by epoxy.

Microstructure parameters that can be directly controlled during the manufacturing process are of particular interest. For HSCs, such parameters include the nominal wall thickness of the hollow spheres and the amount of epoxy within the composite. The influence of other parameters, such as the sphere packing density and the shell size distribution, is also of interest because these parameters could also be regulated, albeit with greater effort. For example, particle-filled hollow spheres could be screened for minimum and maximum diameters to influence the sphere size distribution [11]. A comprehensive understanding of the impact of manufacturing parameters facilitates the identification of production steps that require meticulous attention depending on the sensitivity of the effective stiffness with respect to the parameters influenced by said production step.

We demonstrate that the previously outlined material modeling approach is able to represent industrial HSCs. We utilize computational homogenization methods based on the Fast Fourier Transform (FFT), originally developed by Moulinec and Suquet [52, 53] and subsequently extended in various directions [54–56]. FFT-based methods operate on a regular voxel grid and prove advantageous for complex microstructures such as the HSCs, since boundary-conforming mesh generation is avoided. Determining the material phase is rather straightforward if level-set functions are available, offering a fully contained homogenization framework. Of particular relevance to the work at hand is the use of stable discretizations [57, 58] required to handle porous materials [59] and the composite voxel method [60, 61]. The latter requires determining the volume fractions of the individual material phases within interface voxels, and the provided analytic level-set representation permits determining those fractions in a grid-independent and robust way.

The high damping of HSCs is a geometric effect, improving the dissipation capabilities significantly compared to the dense bulk material. Since the forces between neighboring spheres are transmitted through thin shells and epoxy bridges, a modest macroscopic strain produces rather large strain and stress amplitudes locally in these connective regions, increasing material damping [62, 63]. Predicting the damping of an HSC, therefore, reduces, in essence, to predicting where and how strain is distributed in the microstructure under load. Therefore, any error made for the elastic model is inherited by the identified damping parameters. Following the elastic-viscoelastic correspondence principle [64], we therefore determine the elastic response first, leaving only the dissipative parameters to be identified in a subsequent step. The material modeling in this linear elastic step is the focus of the present work.

In Sect. 2.1, we introduce the microstructure under consideration. Section 2.2 summarizes the experimental approach to determine the effective elastic properties of the material using the impulse excitation technique (IET). In Sect. 2.3, we discuss the morphological closing operator used to determine the connectivity between the spheres within an HSC as well as the general approach to microstructure generation of an HSC. To perform the erosion operation, a level-set function must be determined first. Therefore, in Sect. 2.4, we introduce an algorithm for determining the level-set function of a three-dimensional union of balls and discuss how to identify the material phase for any point within the domain. We also present some resulting periodic microstructures.

In Sect. 4, we study the influence of different factors on the ensuing mechanical properties. To do so, we use the findings obtained in Sect. 3. Furthermore, we determine the local stress fields in Sect. 4.1, where we also analyze the stress localization. In Sect. 4.2, we present the influence of the volume fraction of epoxy, the shell thickness of the individual spheres, the sphere size distribution and the sphere packing density on the elastic moduli. Section 4 concludes the computations with a validation of the computational model by comparing the experimental results from Sect. 2.2 and another composite from the literature. Finally, we draw a conclusion in Sect. 5.

The main contributions of this work are:

(i) a dilation–erosion level-set formulation for glued hollow-sphere assemblies,
(ii) its integration into an FFT-based homogenization framework, and
(iii) the use of composite voxels based on an analytic interface description to accurately resolve thin interphases.

## Microstructure modeling

### Microstructure characterization

We are interested in the microstructure of hollow sphere composites. More precisely, we consider both particle filled and unfilled hollow sphere composites. Due to the particles’ free movement, the filler is not expected to significantly affect the material’s elastic properties. Therefore, we do not distinguish between these two types of composites and refer to both as HSCs.

**Fig. 1:**
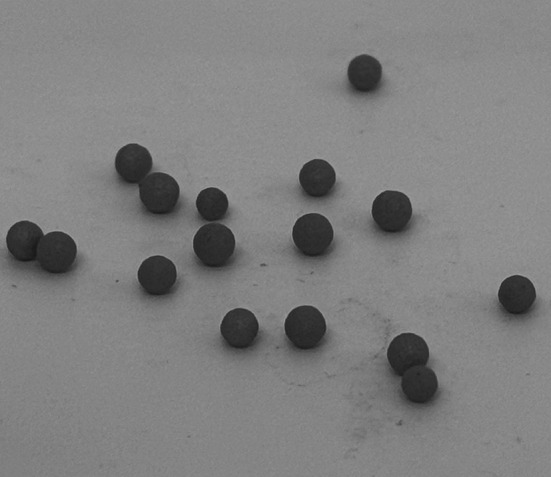
A small sample of HSs

**Fig. 2:**
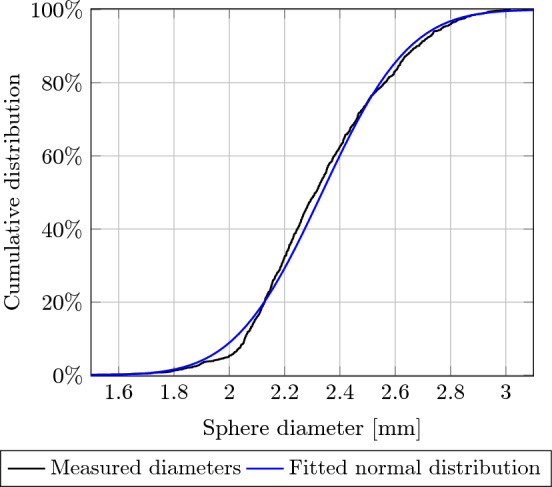
Distribution of the HS diameters

The HSCs internally consist of HS that are adhered with an epoxy. The individual HS are fabricated by coating a small expanded polystyrene (EPS) sphere in a fluid bed reactor. For a start, a first coating, a mixture of Al$_{2}$O$_{3}$ particles, binder, and polymer space holders, is applied. Then, a second coating, a mixture of carbonyl iron powder, with a 4 wt% Fe$_{3}$P additive, and a binder, is applied. The resulting spheres are then debindered through the exposure to steam and evaporated isopropanol, whereby the polymer space holders and the EPS spheres are dissolved as well, s.t. the Al$_{2}$O$_{3}$ particles can move freely in the interior of the HS. Afterwards, the HSs are placed in an oven under a hydrogen atmosphere and normal pressure, where they are further debindered, and then sintered. Finally, the HSs are adhered using a thermally cross-linked adhesive powder (Araldit^®^ AT 1-1) by coating the sintered HSs with a third layer of an epoxy solution in acetone, followed by molding. The mold containing the coated HSs is placed in an oven at $140^\circ$C for two hours to allow the epoxy to cure. A more detailed description of the fabrication process as well as the analysis and discussion of the resulting material properties is given by Jehring [23]. In Fig. 1, a few of the hollow spheres created by the aforementioned process are shown. The target value for the diameter of these spheres was set to 3 mm, but variations in the fabrication process lead to a distribution of the sphere sizes. To determine the actual diameter distribution, the produced HS were scanned and the obtained projections were measured through image processing, which led to the sphere size distribution shown in Fig. 2. The sphere size distribution follows closely the fitted normal distribution with as mean of 2.336 mm and a standard deviation of 0.249 mm. The fitted normal distribution is shown in blue alongside the measured distribution in Fig. 2. Using these HS, a HSC specimen with the nominal spatial dimensions of 300 mm $\times$ 100 mm $\times$ 10 mm was produced to validate the microstructure model presented in this paper.

Previous investigations show that the actual wall thickness of the HS has a major influence on the stiffness [65]. Zhou et al. [66] present a non-destructive method of determining wall thickness and the weight of individual spheres based on statistical modeling. The model was validated by scanning and analyzing the cross-sections of multiple HS, see Fig. 4. Weighing a representative random sample of HS resulted in an average weight of 8.3 mg per HS. Furthermore, the average bulk density of the HS was measured to be 0.66 g/cm$^3$. Subsequently, using the average weight of the HS, we calculated that there are 79.52 spheres per cubic centimeter on average, with an average diameter of 2.336 mm and the associated volume 6.71 mm$^3$. Furthermore, we determined an average wall thickness of 73.9 $\upmu$m. Combining the information of the average weight, the volume, and the bulk density of the HS, we may determine the average sphere packing density of the HS to be 53.3%.

**Fig. 3:**
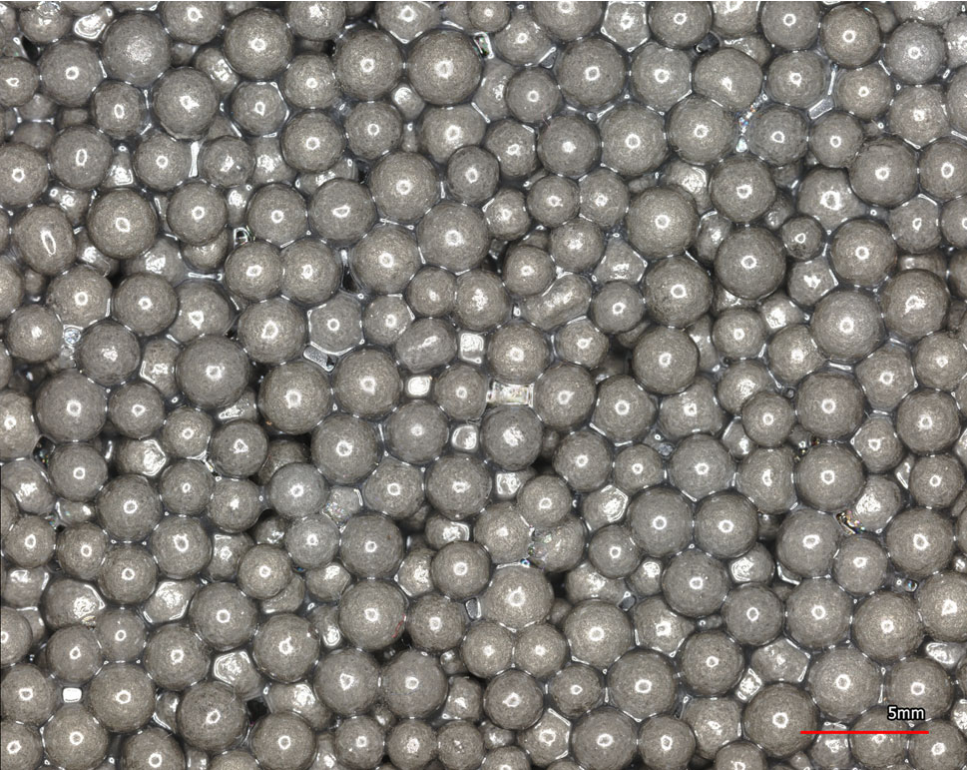
Close-up photograph of a HSC, adhered with a thermally cross-linked epoxy

The mean density of the HSC, see Fig. 3, was measured to be 0.849 g/cm$^3$. The bulk density of the HS is then subtracted from the density of the HSC to calculate the contribution of the epoxy to the density of the HSC. Subsequently, the result is divided by the density of the epoxy (1.16 g/cm$^3$), thus enabling us to determine the volume fraction of 16.2% of epoxy in the HSC.

**Fig. 4:**
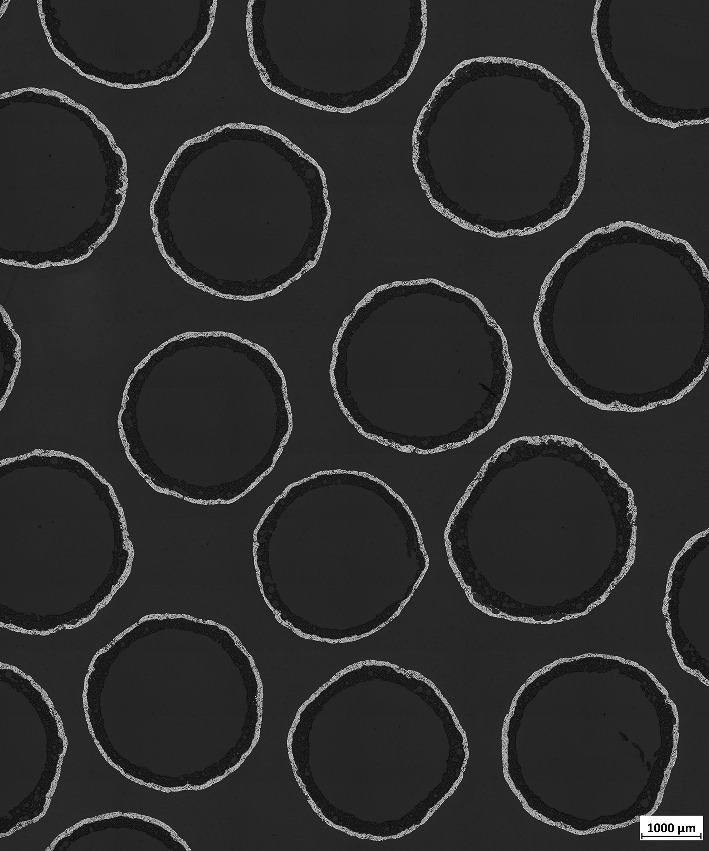
Micrograph of a cross section of multiple HS (scale 1000 $\upmu$m)

### Experimental preinvestigations

For subsequent validation, we report on experimental results of a test specimen that conforms to the specifications in Sect.  2.1. For more detailed descriptions and explanations, we refer to dedicated works [15, 67]. More precisely, the Young’s modulus and the shear modulus were determined via the impulse excitation technique (IET), in accordance with the ASTM standard [67]. To determine the Young’s modulus, a flexure oscillation is excited through an impulse. The flexure resonance frequency $f_f$ of this oscillation is measured and permits to determine the Young’s modulus via the formula

2.1 $$\begin{aligned} E = 0.9465\,\frac{m\, f_f^2}{w}\,a^3\, T_1, \end{aligned}$$

where the aspect ratio $a={\ell }/{t}$ encodes the length $\ell$ and the thickness *t* of the specimen, while *m* represents the specimen’s mass and *w* its width. Moreover, the correction factor $T_1$ for the fundamental flexure must be specified. For specimens with small aspect ratios, i.e., an aspect ratio *a* less than 20, it accounts for the Poisson’s ratio $\nu$ and higher order dependencies of the aspect ratio. As we are only concerned with specimens of higher aspect ratios, a correction factor for the fundamental flexure with no dependencies of the Poisson’s ratio $\nu$ suffices, and we omit the iterative scheme sketched in the ASTM standard [67] to determine the Young’s modulus by using the expression

2.2 $$\begin{aligned} T_1 = 1+6.585\,a^{-2}. \end{aligned}$$

The shear modulus is determined in a similar fashion by exciting a torsional oscillation through an impulse. Measuring the fundamental torsional frequency $f_t$ permits us to determine the shear modulus *G* via the formula

2.3 $$\begin{aligned} G = 4 \, \frac{a\,m\,f_t^2}{w}\left( \frac{B}{1+A}\right) , \end{aligned}$$

where two correction terms *A* and *B* are introduced. Both parameters encode higher-order dependencies on the quotient $b={t}/{w}$ of the thickness and the width, and are given by

2.4 $$\begin{aligned} A = \frac{0.5062b^3 - 0.8776b^2 + 0.3054b - 0.0078}{12.03b^2 + 9.892b} \quad \text {and} \quad B = \frac{b^2+1}{4b^2-2.52b^3+0.21b^7}. \end{aligned}$$

The correction factor *A* is relevant for high accuracies only, as it has an effect of less than $2\%$.

**Fig. 5:**
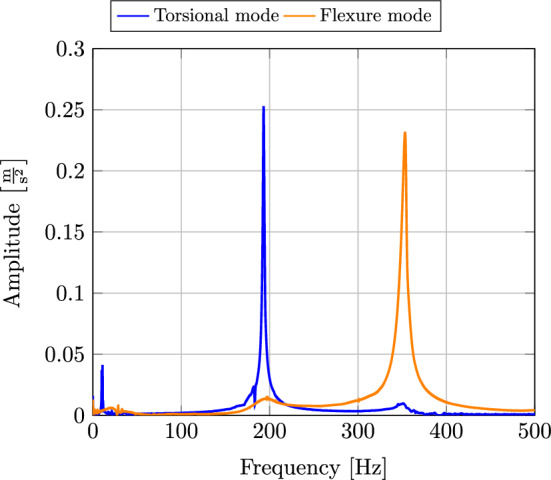
The frequency response for torsional and flexure oscillations of the test specimen

During the test, the specimen described in Sect. 2.1 was excited by an impulse hammer with a steel tip. The response was captured in the time domain at a sampling rate of $19.2\,\text {kHz}$ using an accelerometer mounted on the specimen. The recorded time signal was then transformed into the frequency domain via FFT. The fundamental flexural frequency of $193.33\,\text {Hz}$ and the torsional frequency of $353.21\,\text {Hz}$ were estimated from the peaks in the measured excitation amplitude spectrum, see Fig. 5. With these resonance frequencies and the equations (2.1) and (2.3) at hand, the Young’s modulus is calculated as $E=2.71\,\text {GPa}$, and the shear modulus is obtained as $G=1.12\,\text {GPa}$. Subsequently, the Poisson’s ratio $\nu$ is determined via the known relation, $\nu = 0.5{E}/{G}-1$, as $\nu =0.21$.

### Sphere packing and the addition of epoxy

After identifying the shortcomings of the current state of the art, see Sect. 1.1, our goal is to create sphere packings and connect them with an epoxy phase in a realistic way. We rely on the mathematical description of the union of balls. In this context, it is important to distinguish between the mathematical and colloquial definitions of a sphere and a ball. In mathematical jargon, a sphere refers to the *surface* of a ball, the latter being volumetric. We will use both terms in their respective contexts.

To describe the microstructure, we consider a periodized rectangular cell

2.5 $$\begin{aligned} Y=\left[ 0,L_1\right] \times \left[ 0,L_2\right] \times \left[ 0,L_3\right] , \end{aligned}$$

where $L_i$ ($i=1,2,3$) stand for the edge lengths of the unit cell. Our starting point is a disjoint union

2.6 $$\begin{aligned} B=\underset{i\in \mathcal {I}_B}{\bigcup }b_i \end{aligned}$$

of balls

2.7 $$\begin{aligned} b_i=\left\{ {\boldsymbol{ x}}\in Y\,\big |\, \text {dist}_Y({\boldsymbol{ x}},{\boldsymbol{ c}}_i) \le r_i\right\} \end{aligned}$$

with centers ${\boldsymbol{ c}}_i\in Y$ as well as radii $r_i > 0$, the periodic distance $\text {dist}_Y$ and where

2.8 $$\begin{aligned} \mathcal {I}_B = \{1,2,\ldots ,n\} \end{aligned}$$

denotes the index set of all contained balls. We obtain the sphere packing (2.6) by the SAM-algorithm [41], where we enforce a minimum distance between individual spheres to prevent connections when discretizing the microstructure. We refer to Schneider et al. [68] for a description of the overlap removal in the case of spheres.

To represent the epoxy phase of the HSC, we apply a closing operation to the union of balls. The closing consists of two steps. First, a dilation is applied, which grows the structure by a fixed amount $r_c>0$, called the closing radius, in every direction. Secondly, the erosion phase takes place, which shrinks the resulting structure by the same amount $r_c$. Although the two steps use the same radius, they do not cancel each other: Gaps and narrow channels that the dilation fills in remain filled after the erosion, since the erosion can only peel off material from the current boundary inward. The closing therefore glues together regions that were close to one another in the original assembly, while leaving the bulk shape largely unchanged.

**Fig. 6:**
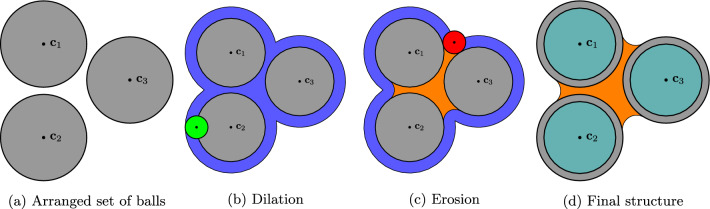
Stages of the construction of the microstructure, with initial disks (grey), additional area obtained by dilation (blue), final glue after erosion (orange), and the core (teal)

Concretely, for a union of balls the dilation step is immediate. Enlarging a union of balls *B* by the closing radius $r_c$ is equivalent to keeping the sphere centers fixed and increasing each radius from $r_i$ to $r_i+r_c$, for all $i\in \mathcal {I}_B$. The erosion step is the harder one, since after dilation the individual balls overlap and the boundary of the union no longer consists of full sphere surfaces. The erosion removes every point whose distance to the boundary is less than the closing radius $r_c$. The leftover material – the part added by the dilation that survives the subsequent erosion – represents the epoxy phase.

A two-dimensional sketch is given in Fig. 6a. It shows the original disks in gray. The blue area in Fig. 6b is the additional area gained by the dilation. The green disk illustrates the dilation visually, moving its center along the entire boundary of the original set, sweeps out a band of width $r_c$, and the dilated set is the union of the original set with this swept band. Figure 6c shows the resulting structure after the erosion. The orange area is what remains of the dilation after points within $r_c$ of the dilated boundary have been removed. The red disk illustrates the erosion in an analogous, sweeping sense. Its center is moved along the entire boundary of the dilated set, sweeping out an inward band of width $r_c$, and this band is then removed. Only those points the sweeping disk never reaches are retained.

In a final step, see Fig. 6d , each ball $b_i$ is assigned an inner radius $r_{\text {in},i} > 0$ to complete the description of the HSC. This inner radius implicitly encodes the wall thickness, and, for apparent reasons, the inequality $r_{\text {in},i} < r_i$ has to hold for all indices $i \in \mathcal {I}_B$ to obtain a non-empty spherical shell. Figure 6 also illustrates that erosion does not undo dilation. The gaps filled by the dilation are not reopened by the erosion. If they were, the closing would have no effect.

### Explicit level-set description of the union of balls

The goal of this section is to determine the signed distance to the surface of a union of overlapping spheres. Since no closed-form expression is available, we construct the distance function using a projection-based approach, which iteratively projects a point onto lower-dimensional geometric features of the structure.

Level-set functions enable us to describe and generate voxel images of microstructures by encoding the smallest distance to the surface. Moreover, level-set functions provide an inclusion/exclusion indication for an arbitrary point $x\in Y$ based on the sign. In other words, if the level-set function is negative, then the point lies within the described set, and vice versa. Additionally, a level-set function can be used to describe an erosion operation. It is also used for the composite voxels, which are introduced in Sect. 3.1. There, it is used to approximate the normal direction of a material interface and the volume fractions of the constituent materials within the composite voxels [69].

As discussed in Sect. 2.3, we determine the epoxy phase in the HSC by applying the closing operation. The dilation step is immediate by increasing the radius of all balls $b_i$, parameterized by the index $i\in \mathcal {I}_B$. The erosion step, however, is more involved: We need to identify the minimum distance to the surface $\partial B$ of the union of balls *B*, see Eq. (2.6). The level-set function, and thus the distance to the surface, is known for individual balls. However, there is no explicit formula for the level-set function of the union of balls. Therefore, we utilize the decomposition of the domain *Y* into Dirichlet (also called Voronoi) cells, discussed in detail in Apx. A, to determine the level set value of a point ${\boldsymbol{ x}}\in Y$. The basic strategy proceeds by projecting a query point ${\boldsymbol{ x}}\in Y$ onto the surface of the union of balls *B*, i.e., by projection it along the boundary of the Dirichlet cell which contains the point ${\boldsymbol{ x}}$. Before we concern ourselves with the mathematical definitions, we motivate the utilized projection scheme.

**Fig. 7:**
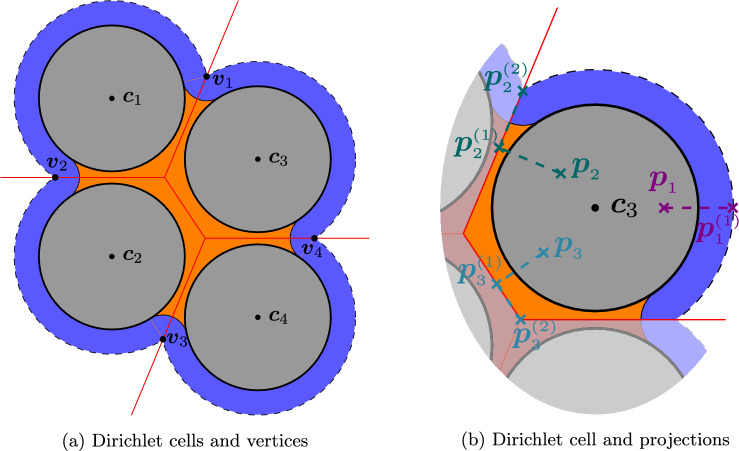
Representation of the Dirichlet tessellation, the resulting vertices, and the projection scheme used to determine the level-set function for a two-dimensional union of four balls

In Fig. 7, we take a look at three generic points ${\boldsymbol{ p}}_1,{\boldsymbol{ p}}_2,{\boldsymbol{ p}}_3\in Y$ whose level-set values we want to determine – that is, the signed distance to the surface of the union of balls. As mentioned, the dilation is simple to compute by increasing the radius of the balls, for this union of balls after the dilation, we aim to determine the level-set function.

We start by considering a Voronoi tessellation on the union of balls, i.e., we divide the domain into cells (one per ball), which are bounded by planes that are equidistant from the surfaces of the balls, see the orange lines in Fig. 7b. Furthermore, we introduce the points ${\boldsymbol{ v}}_i\in \mathcal {V}$, $i=1,\dots ,4$, in the vertex set $\mathcal {V}$, comprised of those points which are elements of two different Dirichlet cells and the surface of the domain.

In Fig. 7b, the Dirichlet cell associated with the ball $b_3$ is shown and the projection paths for the three points ${\boldsymbol{ p}}_1$, ${\boldsymbol{ p}}_2$ and ${\boldsymbol{ p}}_3$ are highlighted. For the point ${\boldsymbol{ p}}_1$, the minimum distance is realized by a radial projection from the center ${\boldsymbol{ c}}_3$ of ball $b_3$ onto the surface after the dilation. Subsequently, the negatively weighted distance between the point ${\boldsymbol{ p}}_1$ and its projection ${\boldsymbol{ p}}_1^{(1)}$ gives us the value of the level-set function.

For the point ${\boldsymbol{ p}}_2$, the projection onto the surface of the ball $b_3$ does not lie within the Voronoi cell. Therefore, we select the closest projection onto a plane of the Voronoi cell. For the point ${\boldsymbol{ p}}_2$, the closest projection onto a plane of the Voronoi cell corresponds to the point ${\boldsymbol{ p}}_2^{(1)}$. However, this is not a radial projection from the center ${\boldsymbol{ c}}_3$, but an orthogonal projection onto the plane in its normal direction. The point ${\boldsymbol{ p}}_2^{(1)}$ still lies within the union of balls. For the next projection, we utilize the fact that an intersection of a *d*-dimensional ball with a plane gives rise to a $(d-1)$-dimensional ball, in our case, the intersection of the plane with the ball $b_3$ gives a 1-dimensional ball – better known as a line segment. Within this lower dimensional ball, we project the point ${\boldsymbol{ p}}_2^{(1)}$ onto its surface. The resulting projected point ${\boldsymbol{ p}}_2^{(2)}$, lies on the surface of the union of balls, and via the negative distance, we again obtain the level-set function.

As a last example, we consider the point ${\boldsymbol{ p}}_3$. Here, the radial projection also lies outside the Voronoi cell. Again, we select the closest, orthogonal projection ${\boldsymbol{ p}}_3^{(1)}$, on a plane of the Voronoi cell. Similar to the point ${\boldsymbol{ p}}_2^{(1)}$, we project ${\boldsymbol{ p}}_3^{(1)}$ on the line segment. This time however, the endpoints of the line segment do not lie within the Voronoi cell. Therefore, our projection within the segment does not lie in the cell and we select the closest projection onto a plane within the line segment, i.e., we obtain the point ${\boldsymbol{ p}}_3^{(2)}$. The point ${\boldsymbol{ p}}_3^{(2)}$ obviously does not deliver the closest distance to the surface of the union of balls since it does not lie on it. To fix this shortcoming, we instead choose the minimum distance between ${\boldsymbol{ p}}_3$ and a vertex ${\boldsymbol{ v}}_i\in \mathcal {V}$, $i=1,\dots ,4$. In this case, the closest point would be ${\boldsymbol{ v}}_4$.

This scheme is generalizable to arbitrary dimensions, where for *d* dimensions a maximum of *d* projections is necessary to determine the closest point on the surface of the union of balls.

To formalize this procedure in three dimensions, we start by defining the projection of the point ${\boldsymbol{ x}}$ onto the surface of a ball $b_i$:

2.9 $$\begin{aligned} \bar{{\boldsymbol{ x}}}^{(1)} = \frac{{\boldsymbol{ x}}-{\boldsymbol{ c}}_i}{\left\| {\boldsymbol{ x}}-{\boldsymbol{ c}}_i\right\| } \, r_i+{\boldsymbol{ c}}_i. \end{aligned}$$

If the point $\bar{{\boldsymbol{ x}}}^{(1)}$ is contained within the same Dirichlet cell as the point ${\boldsymbol{ x}}$, then we already found the point on the surface of the union of balls *B* minimizing the distance to the point ${\boldsymbol{ x}}$. Otherwise, we have to determine the half-space $h^{(1)}\in \mathcal {H}_i$ among the set of active half-spaces $\mathcal {H}_i$, where the orthogonal projection realizes the minimum distance to the point ${\boldsymbol{ x}}$, i.e., the half-space $h^{(1)}$ is defined as

2.10 $$\begin{aligned} h^{(1)} = \underset{h\in \mathcal {H}}{\text {argmin}}~\left| {\boldsymbol{ n}}_h\cdot {\boldsymbol{ x}}- d_h\right| , \end{aligned}$$

where the defining plane of a half-space *h* is given in the Hesse normal form with the normal ${\boldsymbol{ n}}_h$ and the distance $d_h$. We also define the point projected onto the boundary of this half-space:

2.11 $$\begin{aligned} {\boldsymbol{ x}}^{(1)} = {\boldsymbol{ x}}-{\boldsymbol{ n}}_{h^{(1)}}\left( {\boldsymbol{ n}}_{h^{(1)}}\cdot {\boldsymbol{ x}}- d_{h^{(1)}}\right) . \end{aligned}$$

The intersection of the defining plane of the half-space $h^{(1)}$ with the ball $b_i$ corresponds to the disk

2.12 $$\begin{aligned} b_{i}^{(1)} = \text {ext}\left( h^{(1)}\right) \cap b_i. \end{aligned}$$

Since a disk is a two-dimensional ball, we can repeat the projection presented in Eq. (2.9) similarly with the disk $b_{i}^{(1)}$ and the point ${\boldsymbol{ x}}^{(1)}$ to obtain the point

2.13 $$\begin{aligned} \bar{{\boldsymbol{ x}}}^{(2)} = \frac{{\boldsymbol{ x}}^{(1)}-{\boldsymbol{ c}}^{(1)}}{\left\| {\boldsymbol{ x}}^{(1)}-{\boldsymbol{ c}}^{(1)}\right\| } \, r^{(1)}+{\boldsymbol{ c}}^{(1)}, \end{aligned}$$

where the point ${\boldsymbol{ c}}^{(1)}$ equals the center of $b_{i}^{(1)}$ and $r^{(1)}$ stands for the radius of the ball $b_{i}^{(1)}$. Following the same reasoning as before, we either found the point realizing the minimum distance to the surface of the union of balls *B* or we have to determine the half-space $h^{(2)}$ with the minimum distance to the point ${\boldsymbol{ x}}^{(1)}$:

2.14 $$\begin{aligned} h^{(2)} = \underset{h\in \mathcal {H}\backslash \left\{ h^{(1)}\right\} }{\text {argmin}}~\left| {\boldsymbol{ n}}_h\cdot {\boldsymbol{ x}}^{(1)} - d_h\right| . \end{aligned}$$

The intersection $b_{i}^{(2)} = \text {ext}(h^{(2)})\cap b_{i}^{(1)}$ of the defining plane of the half-space $h^{(2)}$ and the ball $b_{i}^{(1)}$ corresponds to a one-dimensional ball, i.e., a line segment. As before, we define the point

2.15 $$\begin{aligned} {\boldsymbol{ x}}^{(2)} = {\boldsymbol{ x}}-{\boldsymbol{ n}}_{h^{(2)}}\left( {\boldsymbol{ n}}_{h^{(2)}}\cdot {\boldsymbol{ x}}- d_{h^{(2)}}\right) \end{aligned}$$

and repeat the projection analogously one last time:

2.16 $$\begin{aligned} \bar{{\boldsymbol{ x}}}^{(3)} = \frac{{\boldsymbol{ x}}^{(2)}-{\boldsymbol{ c}}^{(2)}}{\left\| {\boldsymbol{ x}}^{(2)}-{\boldsymbol{ c}}^{(2)}\right\| } \, r^{(2)}+{\boldsymbol{ c}}^{(2)}. \end{aligned}$$

If the point $\bar{{\boldsymbol{ x}}}^{(3)}$ still does not lie on the surface $\partial B$, then we determine the vertex ${\boldsymbol{ v}}\in \mathcal {V}$ with the minimum distance from the set of vertices $\mathcal {V}$, see Fig. 7. In total, we obtain the projection

2.17 $$\begin{aligned} P_b({\boldsymbol{ x}})&=\left\{ \begin{array}{rl} \bar{{\boldsymbol{ x}}}^{(1)}, &  \bar{{\boldsymbol{ x}}}^{(1)}\in \mathcal {D}_{b_i}\\ \bar{{\boldsymbol{ x}}}^{(2)}, &  \bar{{\boldsymbol{ x}}}^{(2)}\in \mathcal {D}_{b_i}~~\text {and}~~\bar{{\boldsymbol{ x}}}^{(1)}\notin \mathcal {D}_{b_i}\\ \bar{{\boldsymbol{ x}}}^{(3)}, &  \bar{{\boldsymbol{ x}}}^{(3)}\in \mathcal {D}_{b_i}~~\text {and}~~\bar{{\boldsymbol{ x}}}^{(2)}\notin \mathcal {D}_{b_i}\\ \underset{{\boldsymbol{ v}}\in \mathcal {V}}{\text {argmin}}~\left\| {\boldsymbol{ x}}- {\boldsymbol{ v}}\right\| , & \text {otherwise}. \end{array} \right. \end{aligned}$$

The level-set function follows immediately:

2.18 $$\begin{aligned} \Gamma ({\boldsymbol{ x}}) = \left\{ \begin{array}{rl} -\left\| {\boldsymbol{ x}}- P_b({\boldsymbol{ x}})\right\| , &  {\boldsymbol{ x}}\in B\\ \underset{i\in \mathcal {I}_B}{\text {min}}\left\| {\boldsymbol{ x}}-{\boldsymbol{ c}}_i\right\| -r_i, &  \text {otherwise}, \end{array} \right. \end{aligned}$$

where we also handle the case that the point under consideration lies outside the union of balls by computing the distance to all surfaces of the balls and taking the minimum.

The procedure to obtain the level set value is summarized in Algorithm 1. The sets $\mathcal {H}_i$ and $\mathcal {V}$ may be precomputed and stored for a given microstructure.

**Algorithm 1:**
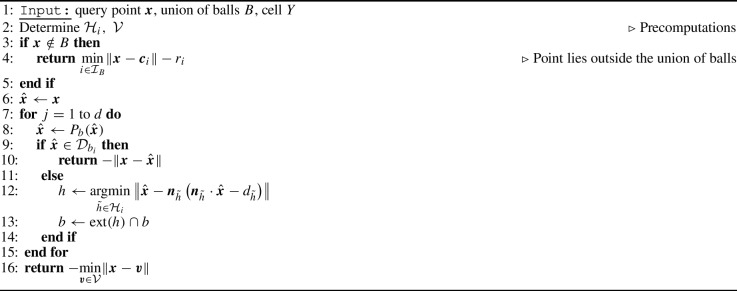
Determining the level set value for a point ${\boldsymbol{ x}}\in \mathcal {D}_{b_i}$

To determine the containing phase for a given point ${\boldsymbol{ x}}$ in the unit cell *Y*, we define the individual indices $I_{\text {core}}$, $I_{\text {shell}}$, $I_{\text {epoxy}}$, and $I_{\text {pore}}$ and assign them via

2.19 $$\begin{aligned} I_{\text {mat}}({\boldsymbol{ x}}) = \left\{ \begin{array}{rl} I_\text {core}, &  {\exists i\in \mathcal {I}_B:~\left\| {\boldsymbol{ x}}-{\boldsymbol{ c}}_i\right\| \le r_i-t_i,}\\ I_\text {shell}, &  {\exists i\in \mathcal {I}_B:~r_i-t_i<\left\| {\boldsymbol{ x}}-{\boldsymbol{ c}}_i\right\| \le r_i,}\\ I_\text {epoxy}, &  {\forall i\in \mathcal {I}_B: ~\left\| {\boldsymbol{ x}}-{\boldsymbol{ c}}_i\right\| >r_i ~~\text {and}~~ \Gamma ({\boldsymbol{ x}})\le -r_c,}\\ I_\text {pore}, & \text {otherwise}. \end{array} \right. \end{aligned}$$

Ultimately, the phase for any point ${\boldsymbol{ x}}$ in cell *Y* is determined by Alg. 2, which heavily relies on Alg. 1 to determine the level set values. Separating the two algorithms improves readability, and Alg. 1 may be used for different purposes, e.g., for computing the data required composite voxels [69].

**Algorithm 2:**
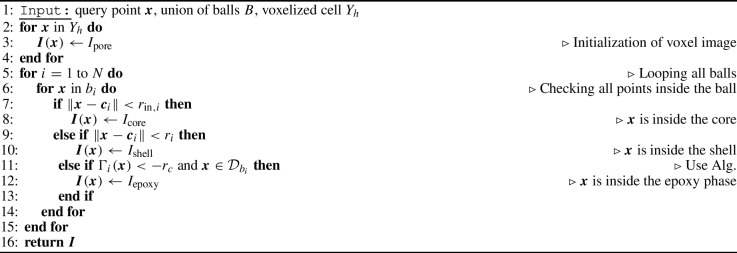
Assigning the material phase to the voxel centers ${\boldsymbol{ x}}\in Y_h$

Some comments are in order:

- A derivation of the level set function for arbitrary spatial dimensions is provided in Apx. B.
- When dealing with periodic structures, we utilize the periodic distance $\text {dist}_Y$ when computing the Dirichlet cell and the level set function. In particular, the concept of periodicity requires considering planes in multiple directions for the same pair of spheres when constructing the Dirichlet cell.
- We motivated the projection (2.17) in a procedural manner. Therefore, it is assured that we obtain the half-spaces $h^{(1)}$, see Eq. (2.10), and $h^{(2)}$, see Eq. (2.14). If, however, the condition $\bar{{\boldsymbol{ x}}}^{(1)}\in \mathcal {D}_{b_i}$ holds, then the procedure for the points $\bar{{\boldsymbol{ x}}}^{(2)}$ and $\bar{{\boldsymbol{ x}}}^{(3)}$ is not defined. To fix this problem, we extend the definition as follows:
  2.20 $$\begin{aligned} \bar{{\boldsymbol{ x}}}^{(i)} = \left\{ \begin{array}{rl} \bar{{\boldsymbol{ x}}}^{(i-1)}, &  \bar{{\boldsymbol{ x}}}^{(i-1)}\in \mathcal {D}_{b_i},\\ \frac{{\boldsymbol{ x}}^{(i-1)}-{\boldsymbol{ c}}^{(i-1)}}{\left\| {\boldsymbol{ x}}^{(i-1)}-{\boldsymbol{ c}}^{(i-1)}\right\| }r^{(i-1)}+{\boldsymbol{ c}}^{(i-1)}, &  \text {otherwise}, \end{array} \right. \quad \text {for}\quad i=2,3. \end{aligned}$$
- To adjust the volume fraction of the epoxy, the closing radius $r_c$ is altered. To determine the radius which realizes the desired volume fraction of the epoxy phase, we alter the closing radius according to an iterative scheme, see Ravichandran et al. [70, §3.2].
- The case ${\boldsymbol{ x}}\notin B$ in Alg. 1 does not occur for the determination of the phases as described in Alg. 2. More precisely, in Algorithm 2, we iterate over all points within the ball, s.t. the case ${\boldsymbol{ x}}\notin B$ is evaded. Instead, all points not contained in any ball are assigned the porous material by default.

**Fig. 8:**
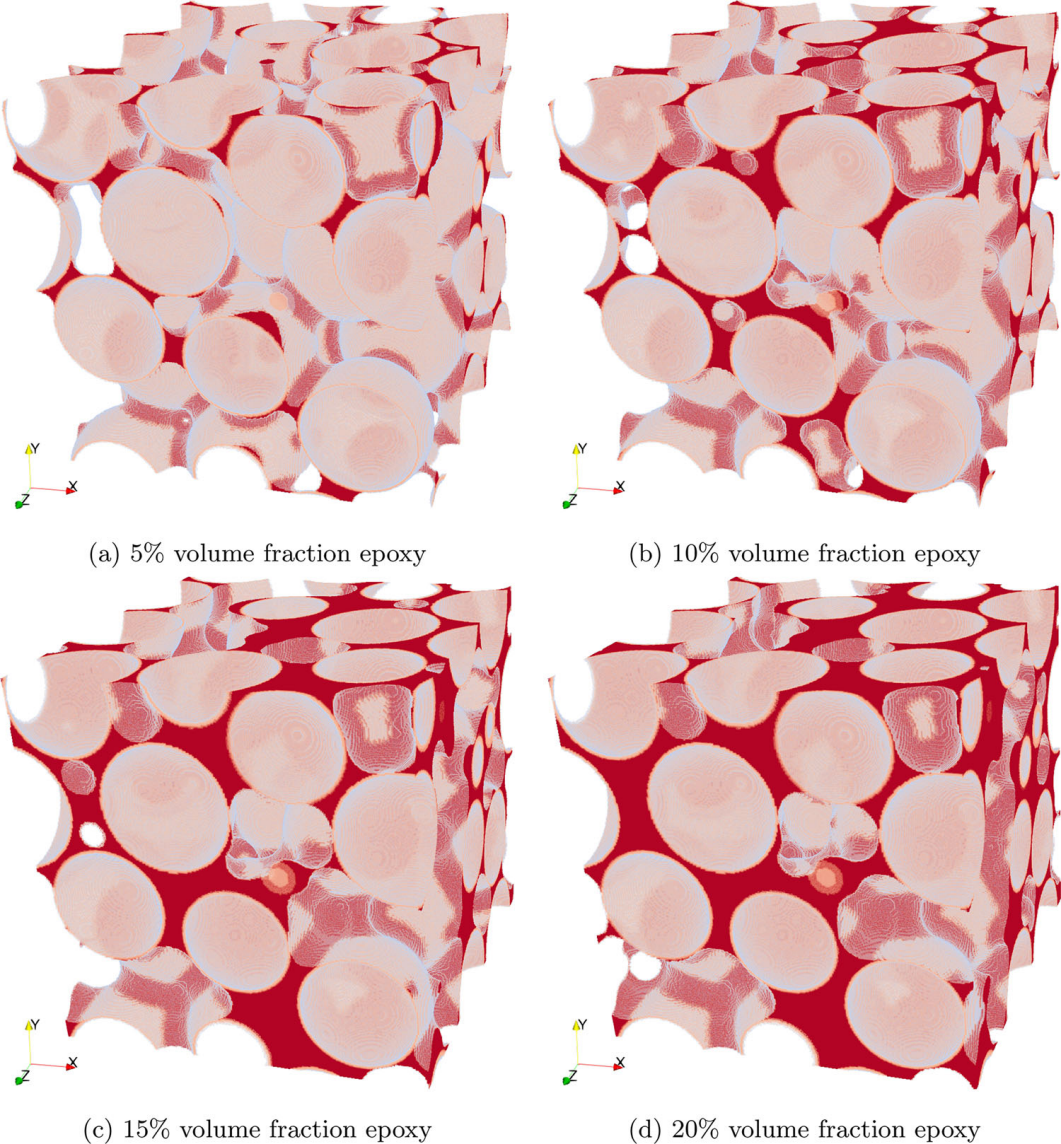
Varying fraction of epoxy between identically arranged HS

After motivating and introducing the microstructure generation, we illustrate the resulting structures in Fig. 8. Within this series of microstructures, we increased the volume fraction of epoxy in $5\%$ steps, from 5 up to 20%. Apart from this change, we keep the microstructure identical: Each one comprises 27 spheres, with a diameter of 2.85 mm, a shell thickness of 72 $\upmu$m and a sphere packing density of $54\%$. We make the following three observations: First, as the volume fraction increases, new connections form between the spheres, as shown in Fig. 8a, b. Secondly, existing connections thicken - compare any of the microstructures in Fig. 8. Third, as the volume fraction increases, the individual connections form larger structures. In Fig. 8d, the epoxy phase represents an interconnected structure.

## Computational micromechanics

### FFT-based methods and composite voxels

We are concerned with a three-dimensional rectangular domain (2.5). For all points in the domain *Y*, we assume a known stiffness distribution

3.1 $$\begin{aligned} \mathbb {C}:Y\rightarrow L(\text {Sym}(3)) \end{aligned}$$

to be given, where $\text {Sym}(3)$ denotes the vector space of symmetric $3\times 3$ tensors and *L*(*A*) represents the linear transformations on the vector space *A*. In the article at hand, we assume that the stiffness distribution $\mathbb {C}$ at every material point ${\boldsymbol{ x}}\in Y$ is determined by the material assigned to the point ${\boldsymbol{ x}}$ via the microstructure modeling detailed in Sect.  2.3. Since the considered material is highly porous, we drop the common requirement of the stiffness tensors to be non-degenerate [59], and instead restrict the computations to the staggered grid discretization [58].

Ultimately, we aim to determine the periodic microscopic strain ${\boldsymbol{\varepsilon }}$ in all points of the unit cell *Y* for a prescribed macroscopic strain $\bar{{\boldsymbol{\varepsilon }}}\in \text {Sym}(d)$, which fulfills the kinematic compatibility condition

3.2 $$\begin{aligned} {\boldsymbol{\varepsilon }}= \bar{{\boldsymbol{\varepsilon }}} +\nabla ^s {\boldsymbol{ u}} \end{aligned}$$

for a periodic displacement fluctuation field ${\boldsymbol{ u}}$, where $\nabla ^s$ denotes the symmetrized gradient. In addition, the equilibrium condition

3.3 $$\begin{aligned} \text {div }{\boldsymbol{\sigma }}= \boldsymbol{0} \end{aligned}$$

must be satisfied, where the constitutive law provides the relationship between the local strain field ${\boldsymbol{\varepsilon }}$ and the local stress field ${\boldsymbol{\sigma }}$ through the Hooke’s law

3.4 $$\begin{aligned} {\boldsymbol{\sigma }}= \mathbb {C}:{\boldsymbol{\varepsilon }}. \end{aligned}$$

By inserting the compatibility condition (3.2) and the constitutive law (3.4) into the equilibrium condition, Eq. (3.3), we obtain an equation

3.5 $$\begin{aligned} \text {div }\mathbb {C}:\left( \bar{{\boldsymbol{\varepsilon }}} + \nabla ^s {\boldsymbol{ u}}\right) = \boldsymbol{0} \end{aligned}$$

for the periodic displacement fluctuation field ${\boldsymbol{ u}}$ only. The solutions of this PDE (3.5) for varying macroscopic strains $\bar{{\boldsymbol{\varepsilon }}}$ allow us to determine the effective material behavior by the implicit definition of the effective stiffness for a representative microstructure

3.6 $$\begin{aligned} \mathbb {C}^{\text {eff}}:\bar{{\boldsymbol{\varepsilon }}} = \left\langle \mathbb {C}:{\boldsymbol{\varepsilon }}\right\rangle _Y, \end{aligned}$$

where $\left\langle \cdot \right\rangle _Y$ denotes the volume average over the domain *Y*. For a fixed homogeneous reference stiffness $\mathbb {C}^0$, the differential form of the problem Eq. (3.5) is equivalent to the Lippmann-Schwinger equation [71, 72]

3.7 $$\begin{aligned} {\boldsymbol{\varepsilon }}= \bar{{\boldsymbol{\varepsilon }}} - \mathbb {\Gamma }^0:\left( \mathbb {C}-\mathbb {C}^0\right) :{\boldsymbol{\varepsilon }} \end{aligned}$$

with the Eshelby-Green operator [73]

3.8 $$\begin{aligned} \mathbb {\Gamma }^0 = \nabla ^s\left( \text {div }\mathbb {C}^0:\nabla ^s\right) ^{-1}\text {div }. \end{aligned}$$

For the latter, explicit representations in Fourier space are known for various discretizations [56]. The suggestive fixed point form of the Lippmann–Schwinger Eq. (3.7) leads to the iterative scheme

3.9 $$\begin{aligned} {\boldsymbol{\varepsilon }}^{k+1} = \bar{{\boldsymbol{\varepsilon }}} - \mathbb {\Gamma }^0:\left( \mathbb {C}-\mathbb {C}^0\right) :{\boldsymbol{\varepsilon }}^{k}, \end{aligned}$$

called the basic scheme. Discretizing the Fourier series via the discrete Fourier transform, Moulinec and Suquet [52, 53] introduced the first discretization scheme used for the FFT-based homogenization methods. Later on, improved discretization methods, more efficient solvers and other extensions were introduced. For a more detailed discussion, we refer to the review article [56].

Composite voxels represent one family of extensions which aim to increase the computational accuracy while maintaining the resolution of a microstructure on a voxel grid [60, 61]. The basic idea of the composite voxel method is to utilize the accessible microstructure information on a sub-voxel level. More precisely, modeling the microstructures analytically enables determining the material interfaces exactly. This piece of information, however, is lost to some degree when discretizing the microstructure on a voxel grid. To mitigate this loss, mixing rules are applied in the voxels containing a material interface: The mixing rule gives rise to a special material law specific to the composite voxel at hand. The individual types of composite voxels differ in the applied mixture rule and/or the procedure to determine the interface [69, 74, 75]. This approach is particularly advantageous when the feature size of the microstructure and the voxel size are of the same order of magnitude, i.e., in the case of the considered microstructure, we are able to better handle the thin shells. By taking into account all adjacent voxels that intersect the geometric surface, the connectivity of the structure is represented with higher fidelity at coarser resolutions.

For composite voxels, using the Voigt/Reuss bounds [60] as mixture rules, the orientation of the interface is neglected and solely the volume fractions of the constituent materials within the voxel are utilized in the mixing. The underlying idea is to assume the strain or stress field of the volume cell to be uniform. For the Voigt bound, we assume that the condition ${\boldsymbol{\varepsilon }}({\boldsymbol{ x}}) = \bar{{\boldsymbol{\varepsilon }}}$ holds for the strain field in all points ${\boldsymbol{ x}}\in Y$. Subsequently, by inserting this assumption into Eq. (3.6), we obtain the effective stress

3.10 $$\begin{aligned} \overline{{\boldsymbol{\sigma }}}&= \left\langle \mathbb {C}:\bar{{\boldsymbol{\varepsilon }}}\right\rangle _Y = \left\langle \mathbb {C}\right\rangle _Y:\bar{{\boldsymbol{\varepsilon }}} = \underbrace{\left( {\boldsymbol{ c}}_1\mathbb {C}_1 + (1-{\boldsymbol{ c}}_1)\,\mathbb {C}_2\right) }_{=\mathbb {C}_{\text {Voigt}}}:\bar{{\boldsymbol{\varepsilon }}}, \end{aligned}$$

where the stiffnesses $\mathbb {C}_1$ and $\mathbb {C}_2$ represent the two stiffnesses of the constituent materials and the associated volume fractions read ${\boldsymbol{ c}}_1$ and ${\boldsymbol{ c}}_2=1-{\boldsymbol{ c}}_1$, respectively. This assumption ultimately gives rise to an upper bound on the stiffness, the Voigt bound $\mathbb {C}_{\text {Voigt}}$ [76, §6].

If, on the other hand, we assume the stress field to be uniform, i.e., ${\boldsymbol{\sigma }}({\boldsymbol{ x}}) = \overline{{\boldsymbol{\sigma }}}$ holds for all ${\boldsymbol{ x}}\in Y$, the Reuss bound emerges. We obtain the effective strain

3.11 $$\begin{aligned}&\bar{{\boldsymbol{\varepsilon }}} = \left\langle \mathbb {C}^{-1}:\overline{{\boldsymbol{\sigma }}}\right\rangle _Y = \left\langle \mathbb {C}^{-1}\right\rangle _Y:\overline{{\boldsymbol{\sigma }}} = \underbrace{\left( {\boldsymbol{ c}}_1\mathbb {C}_1^{-1}+\left( 1-{\boldsymbol{ c}}_1\right) \mathbb {C}_2^{-1}\right) }_{=\mathbb {C}_{\text {Reuss}}^{-1}}:\overline{{\boldsymbol{\sigma }}},\end{aligned}$$

3.12 $$\begin{aligned} \Rightarrow \quad&\mathbb {C}_{\text {Reuss}} = \left( {\boldsymbol{ c}}_1\mathbb {C}_1^{-1}+\left( 1-{\boldsymbol{ c}}_1\right) \mathbb {C}_2^{-1}\right) ^{-1}, \end{aligned}$$

where the Reuss bound $\mathbb {C}_{\text {Reuss}}$ represents a lower bound for the stiffness for the mixing of two phases.

In both cases, the Voigt and the Reuss bounds, we need to determine the volume fractions of the constituent materials. Utilizing the level-set function (2.18), we can obtain a linear approximation of the interface and the volume fractions of the constituent materials in accordance with Lendvai and Schneider [69]. This approach leads to stiffnesses for the composite voxels, which can be computed through a laminate formula. These so-called laminate composite voxels usually offer a better computational accuracy [69]. However, this does not hold true for degenerate constituent materials. In particular, the voids in the microstructure at hand, see Fig. 8, represent a material with a degenerate stiffness tensor. Similarly, the Reuss bound vanishes in case one of the stiffnesses $\mathbb {C}_1$ or $\mathbb {C}_2$ vanishes. Thus, we utilize Voigt composite voxels for the computations at hand, where due to the previously discussed mixing rule, see equation (3.10), the resulting stiffness is non-degenerate even if one constituent material is a void.

### Setup

In this section, we investigate the mechanical properties of the HSC and the influence of the parameters of the microstructure on the mechanical properties on digital images which we generate with the algorithm introduced in Sect. 2. Therefore, we set up a series of computations that align with the findings of Sect. 3.4. Following the discussion of Sect. 3.1, the composite voxel type used for these computations are Voigt composite voxel, see Eq. (3.10). To solve the micromechanical problem, an existing FFT-based computational micromechanics code [77], written in Python with Cython extensions and parallelized with OpenMP, was utilized. We use the conjugate gradient method [78–80] and the staggered grid discretization [58] to solve the linear systems associated with the porous microstructures. For the convergence criterion of the FFT-scheme, we rely on the criterion presented in Schneider et al. [56], where the threshold of $5\cdot 10^{-4}$ proved to be the best compromise between accuracy and run time. The computations were run on a workstation equipped with two AMD^®^ EPYC^™^ 9354 and 1.12TB of RAM.

To determine the effective stiffness $\mathbb {C}^{\text {eff}}$, see equation (3.6), we prescribe six linearly independent macroscopic strains $\bar{{\boldsymbol{\varepsilon }}}_i$, with $i=1,\dots ,6$, giving rise to the system of linear equations: $\left\langle {{\boldsymbol{\sigma }}}\right\rangle _{Y,i} = \mathbb {C}^{\text {eff}}:\bar{{\boldsymbol{\varepsilon }}}_i$. To compare the computational results with experimental data, we make use of a conventional isotropic approximation of the elasticity tensor [81].

### Resolution study

Before we determine the mechanical properties, we first evaluate the response of the introduced model w.r.t. the resolution of the microstructure. Such a study ensures that the error of the resulting stiffness is within a reasonable margin. More precisely, our goal is to determine the number of voxels necessary to represent the geometric structures of the microstructure with sufficient accuracy to provide meaningful results. On the other hand, the resolution should be coarse enough to allow for efficient computations. For the microstructure discussed in this paper, the shells of the HS are the critical elements, as they are particularly thin: For the HSC introduced in Sect. 2.1, the aspect ratio of the shell to the average diameter of the HS is approximately 31.61.

**Table 1:** Material parameters considered for the computational experiments

Sintered steel	$E=140$ GPa,	$\nu =0.3$,	[13] [82]
Epoxy	$E=3.78$ GPa,	$\nu =0.35$,	[83]

To determine the number of voxels required to adequately resolve the HS in the radial direction, a series of computations were conducted. For these computations, the microstructure was fixed, i.e., the centers and radii of the HS and the epoxy volume fraction were kept constant, but the resolution was varied. In addition, we also study the influence of the composite voxels on the precision and subsequently compare the results to the same microstructures utilizing composite voxels with the ones using plain voxels. As no analytical solution is available for the apparent stiffness of the considered microstructure, a high fidelity computation with a resolution of $1024^3$ voxels and active Voigt composite voxels used as a reference. In all computations, the microstructure under consideration contains 27 spheres.

**Fig. 9:**
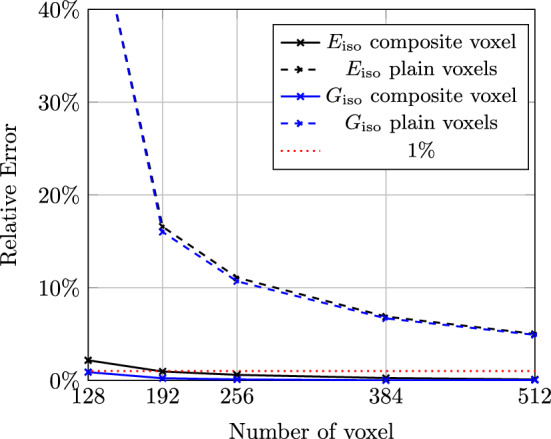
Discretization errors of the isotropic moduli at different resolutions for plain voxels and composite voxels

In Fig. 9, the relative error of the Young’s and the shear modulus or for the isotropic approximation are compared to the moduli of the high fidelity computation for various resolutions. The difference in the relative error between the computations using composite voxels to the ones using plain voxels is immediately visible. The composite voxels only deliver results with a relative error exceeding $1\%$, the defined threshold, for resolutions of $128^3$ voxels. In contrast, the computations with plain voxels never produce results with a fidelity below this threshold, but instead still show an error of greater than $5\%$ for the resolution $512^3$. At this resolution, the shell is resolved by approximately 4.3 voxels across its thickness. For both cases, the computations employing composite and plain voxels, the convergence under grid refinement is clearly evident, and the errors associated with the approximated Young’s and shear moduli are closely aligned.

As we set the threshold for the discretization error to $1\%$, we observe that the computations using plain voxels offer no sufficient precision for reasonable resolutions. Subsequently, we use Voigt composite voxels for our computations, where, for a resolution of $192^3$ voxels, the discretization error first drops below $1\%$ for both, the Young’s and the shear modulus. For the considered microstructure, a resolution of $192^3$ voxels translates into an edge length of approximately $44.35\,\upmu$m, which means that the ratio of the shell thickness to the edge length is equal to approximately 1.62. Consequently, we adopt the rule of thumb that the shell of the HS should be resolved with 2 (composite) voxels over its thickness to ensure a sufficiently accurate result.

**Fig. 10:**
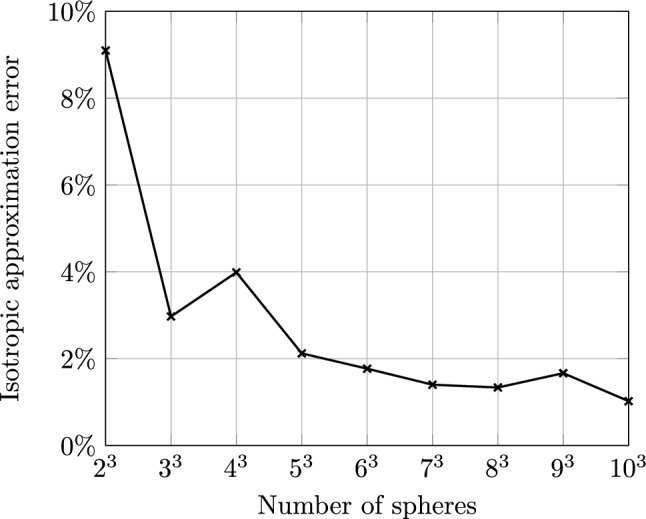
The error of the isotropic stiffness approximation in comparison with the computed stiffness

### Investigating representativity

The investigations in the previous subsection were aimed at determining the resolution required to restrict the discretization error for a fixed microstructure. However, these do not provide information whether the computed apparent properties of the microstructure are close to the effective stiffness, and if the assumed isotropy of the microstructure actually holds. The assumption that the material under consideration is isotropic originates from the absence of a discernible preferred direction, since the HS are arranged randomly. To validate the isotropy assumption, we examine the relative error between the apparent stiffness and its isotropic approximation for a number of differently sized volume elements, which therefore contain a varying number of spheres. The resulting error for the isotropic approximation is displayed in Fig. 10, where the tendency for reduced relative errors at an increased number of HS, and therefore cells with larger volume, is observed. However, there are two data points, namely, for $4^3$ and $9^3$ HS, where the error increased in comparison with the next smaller volume cell. This behavior, however, is likely attributed to statistical variances, as only one simulation was run for each data point. To conclude, the demonstrated decrease in approximation error supports the assumption of isotropy.

**Fig. 11:**
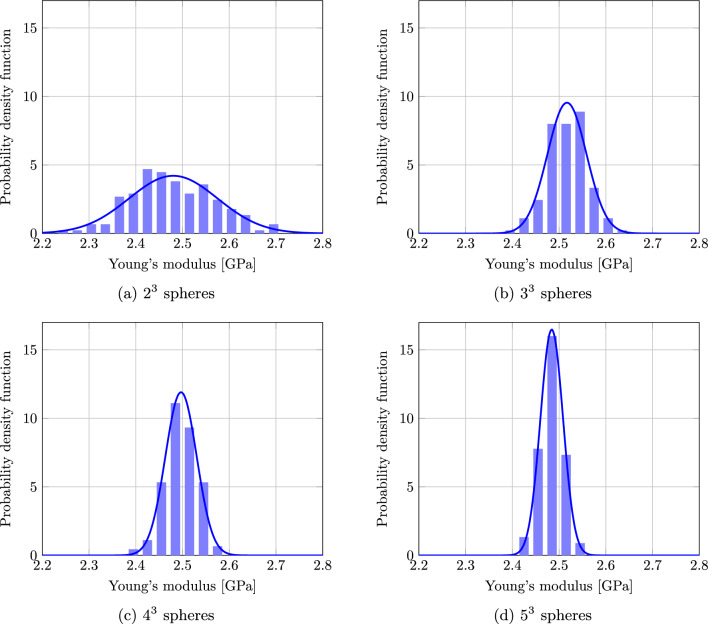
Influence of the volume cell size on the resulting stiffnesses in the ensemble

At this step in the analysis, we are focused on finding the best compromise between representativity and the size of the volume cell. To elaborate, we aim to identify the smallest possible cell volume for which the resulting stochastic error is sufficiently small, thereby requiring only a limited number of computations, and where the computational effort is comparably low, at the same time. For this investigation, we ran 150 computations for four different volume element sizes. Namely, we investigate volume cells with $2^3$, $3^3$, $4^3$, and $5^3$ HS. For all microstructures, the volume fraction of the epoxy phase is fixed at $15\%$, the shell thickness is fixed at 72 $\upmu$m and the sphere packing density of $54\%$ is studied. Following the results of the resolution study, we set the resolution to $256^3$ voxels for the microstructures containing $2^3$ and $3^3$ HS, for the microstructure containing $4^3$ and $5^3$ HS, we chose a resolution of $384^3$ to ensure that the quotient of the shell thickness and the voxel size stays at approximately 2, see Table 2. The resulting distributions of the Young’s modulus of the approximated isotropic stiffness are shown in Fig. 11. The associated stochastic parameters are presented in Table 2. From the visual representation as well as from the stochastic parameters, we deduce that for an increasing size of the volume cell, the standard deviation also decreases. Moreover, the $95\%$-confidence interval of the mean is smaller for larger volume cells, but centered around approximately $2.5\text {GPa}$ for all volume cell sizes. However, the mean value seems to present a tendency to decrease slightly for larger volumes. This phenomenon is probably caused by a reduction of the systematic error, see Schneider et al. [68] for an in-depth discussion, and an increasing discretization error, in case the microstructure is discretized with the same number of voxels, but has different sizes. More precisely, we resolved the volume cells containing $2^3$ and $3^3$ HS with the same resolution; therefore, the individual HS within volume cells containing $3^3$ HS was resolved more coarsely. Since difficulties arise in resolving the shell at coarse resolutions and the surrounding void phase provides no structural support, we expect the stiffness to decrease for HS with coarser resolutions. However, the Young’s modulus increases. Thus, the reduction of systematic error likely outweighs the increase in discretization error. In comparison, the pair of microstructures containing $4^3$ and $5^3$ HS demonstrates an inverse trend, wherein the coarser resolution also results in a lower Young’s modulus. Overall, the microstructure containing $3^3$ HS seems to fulfill the set compromise requirement the best, as the distribution of its Young’s modulus is significantly narrower, while also improving upon the systematic error in comparison with the microstructure containing $2^3$ HS. In addition, the necessary resolution does not exceed $256^3$ voxels, thus offering a significant advantage in the computational effort, since the latter scales roughly linearly with the number of voxels $N^3$. We present a comparison of different runtimes in Table 2. Besides the number of HS, the computations presented in Fig. 11 reveal that it is imperative to utilize more than one computation to determine the material behavior for a certain parameter set of the HSC.

**Table 2:** Parameters of the fitted normal distribution shown in Fig. 11

	$2^3$ spheres	$3^3$ spheres	$4^3$ spheres	$5^3$ spheres
Mean $E_{\text {iso}}$ in GPa	2.480	2.517	2.497	2.484
Standard deviation $E_{\text {iso}}$ in GPa	0.0948	0.0418	0.0335	0.0242
$95\%$-confidence interval $E_{\text {iso}}$ in GPa	[2.465, 2.496]	[2.510, 2.524]	[2.492, 2.502]	[2.480, 2.488]
Minimum $E_{\text {iso}}$ in GPa	2.234	2.406	2.407	2.416
Maximum $E_{\text {iso}}$ in GPa	2.842	2.634	2.577	2.540
Resolution	$256^3$	$256^3$	$384^3$	$384^3$
Voxels per thickness	3.247	2.165	2.435	1.948
Computational time in s (1 thread, 1 loadcase)	1295	1626	4779	6960

## Computational investigations

### Local fields

The local stress fields help to gain a more precise understanding of the inner workings of a material, in our case the HSC. Even when restricting to linear elastic load cases, the resulting localized stress concentrations often act as a precursor to material failure. Therefore, analyzing the local stress distribution for different parameter sets may provide ideas to improve upon, for example, the fatigue resistance and the understanding of failure mechanisms.

**Fig. 12:**
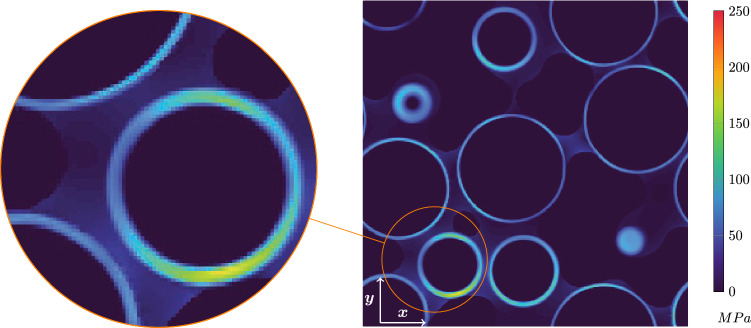
Von Mises stress for a prescribed strain ${\boldsymbol{\varepsilon }}= 0.01\,{\boldsymbol{ e}}_x\otimes {\boldsymbol{ e}}_x$, within a slice in the *x*–*y* plane of the same HSC structure, and a resolution of $256^3$ voxels

For our HSC of interest, described in Sect. 2.1, the local von Mises stress fields are shown in Fig. 12. The microstructure contains $3^3$ spheres, discretized by $256^3$ voxels with active Voigt composite voxels, and the material parameters described in Table 1 were employed. The stress field displays peaks in parts of the shells of the HS where no epoxy is present. We observe that, in general, the majority of the stress loading occurs in the shells. Furthermore, in the presented slice, there is one HS in the lower left-hand corner where the stress is significantly higher than for the other HS, i.e., we observe specific HS which experience significantly higher loads. A zoomed view of this area is located on the left side. Moreover, we observe the comparably small von Mises stress within the epoxy phase. However, the epoxy phase still shows a strong relative variance, e.g., the stress within the epoxy phase on the upper right-hand side of the slice is very small, so it is hard to distinguish the epoxy phase from the void phase. Meanwhile, in the lower left-hand corner and in the zoom view, the epoxy phase also shows elevated levels of stress, and therefore, the shape of the epoxy phase is clearly recognizable.

### Parameter studies

For the following studies, the effective stiffness is evaluated by prescribing six linearly independent strain loads. As shown in Fig. 10, the effective stiffness is isotropic to engineering accuracy. For this reason, we approximate the apparent stiffnesses determined in the individual computations by an isotropic stiffness, which is uniquely determined by the Young’s and the shear modulus. For all computations, composite voxels are utilized.

**Fig. 13:**
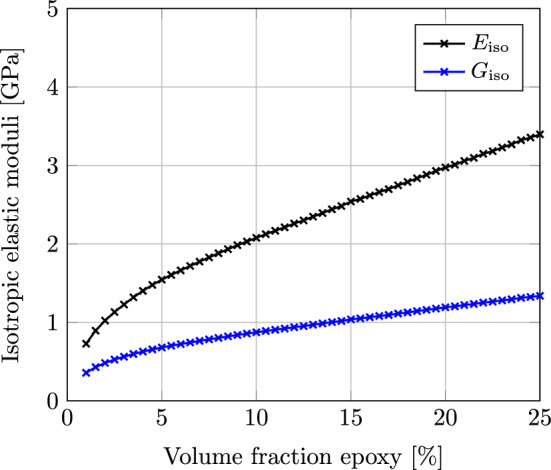
Influence of the volume fraction of the epoxy phase on the Young’s and the shear modulus

**Fig. 14:**
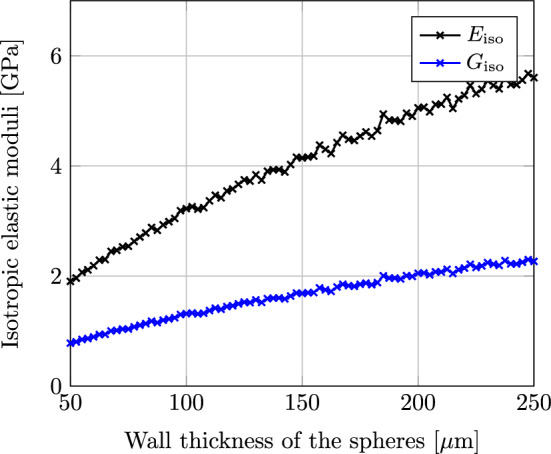
Influence of the wall thickness on the isotropic Young’s and shear modulus approximation

**Influence of the volume fraction of epoxy:** The volume fraction of the epoxy phase is directly influenced by the production process, and can be chosen rather freely. Therefore, its influence on the effective properties is of interest. For this purpose, we set up a microstructure with a sphere packing density of $53\%$, a constant sphere radius of 2.85 mm, and a shell thickness of 72 $\upmu$m. The arrangement of the spheres remains identical for all computations to minimize the influence of the sphere packing. Solely the volume fraction of the epoxy phase is altered. The microstructure – which contains 27 spheres—is resolved by $256^3$ voxels.

The computationally determined influence on the Young’s and shear modulus is presented in Fig. 13, where both curves show parallel trends. Namely, they follow a linear trend for higher volume fractions of epoxy. However, For a volume fraction of epoxy below $5\%$, the curves show a pronounced nonlinearity. Here, the HS still form new connections between each other. These newly formed load paths lead to a higher increase in the stiffness of the material rather than the thickening of the already existing load paths, which is the sole mechanism present for higher volume fractions, see Fig. 8.

**Influence of the shell thickness:** Similar to the volume fraction of the epoxy, the nominal shell thickness can be chosen rather freely. Hence, the dependence of the stiffness of the resulting HSC on the (nominal) shell thickness is of immediate interest. Therefore, we set up a series of computations with fixed sphere packing densities and a constant sphere diameter of 2.85 mm and varying shell thicknesses. Put differently, the inner radius of the shell gets varied, while the outer radius of the shell stays constant. Each HSC used in the computations contained 27 spheres. Special care had to be taken for shell thicknesses smaller than 72 $\upmu$m, as the resolution of the microstructure could possibly be too coarse. Accordingly, we selected the resolution as $512^3$ voxels for microstructures with a shell thickness smaller than 72 $\upmu$m. For all other microstructures, the resolution was set to $256^3$. To reduce the error, we computed the apparent stiffness of two independently generated microstructures for each shell thickness.

The relationship between the obtained elastic moduli and the sphere size is shown in Fig. 14, where the two curves show a similar trend. However, the elastic moduli show a slight nonlinearity for increased sphere sizes, with a steady decrease in the gradient. As the inner radius decreases linearly, the volume fraction of the shells increases nonlinearly. Also, the influence of the shell thickness on the stiffness is quite strong; Over the shown range of the shell thickness, both the Young’s and the shear modulus almost triple.

**Fig. 15:**
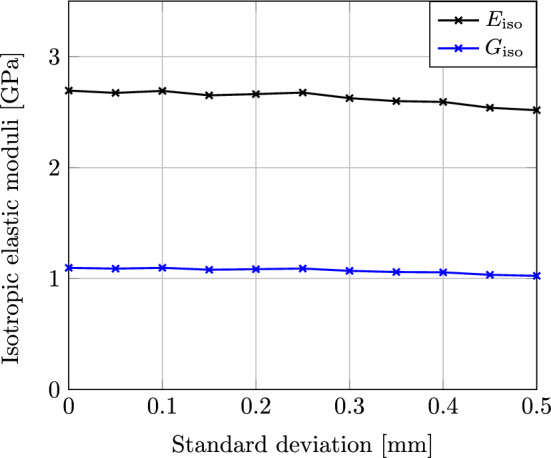
Influence of the standard deviation of the sphere sizes on the isotropic moduli

**Fig. 16:**
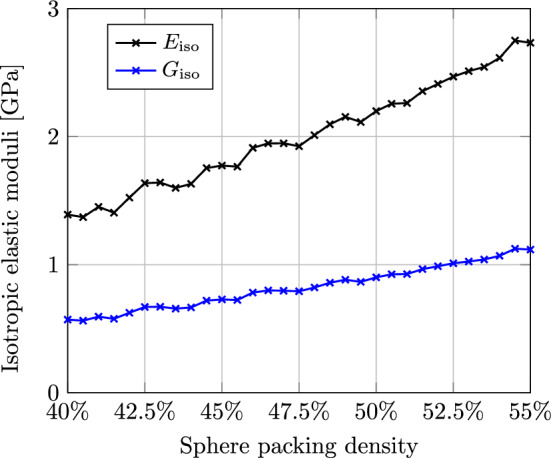
Influence of the sphere packing density on the Young’s and the shear modulus

**Influence of the sphere size distribution:** The production process of the HS leads to varying sphere diameters. These diameters can be measured and are therefore available for as an input for the simulations. In Fig. 2, we observed that the sphere size distribution follows a normal distribution in close fashion. However, we wish to study how this diameter distribution influences the apparent stiffness, and whether measures for controlling the sphere sizes, e.g., improving the production process, are warranted.

For this reason, we analyzed the influence of the sphere size distribution on the stiffness by prescribing normal distributions for the sphere size distribution with a mean of 2.85 mm and varying standard deviation. The microstructures for these computations are resolved by $384^3$ voxels and contain $5^3$ spheres. The shell thickness of each HS is set to 76 $\upmu$m. For each value of the standard deviation, ten computations were evaluated.

The influence of the standard deviation of the sphere size distribution on the apparent stiffness is displayed in Fig. 15. We observe that the influence is marginal. Both the Young’s and the shear modulus show rather small changes for an increasing standard deviation. This observation is interesting because, while we keep the shell thickness constant, the volume fraction of the HS shells is not constant, but depends nonlinearly on the sphere diameter.

**Influence of the sphere packing:** The sphere packing density of HSs may be measured after the production process is completed. However, it cannot be set arbitrarily during production. In Sect. 2.1, the sphere packing density is determined indirectly through the bulk density. Despite the inability to adjust the sphere packing density freely, it is advantageous to understand its influence, for instance, to determine the necessity of measures which ensure that the sphere packing density for varying batches is attained in a reproducible manner. One potential approach involves facilitating the settlement of HS through vibrations, thereby ensuring the consistency of density across batches. This methodology could also be employed to increase the sphere packing density, in case the material properties for the HSC are improved accordingly.

The evaluated computations, ten per data point, resolve the microstructure by $256^3$ voxels and were performed using a sphere with a diameter of 2.85 mm and a shell thickness of 72 $\upmu$m. As shown in Fig. 16, the resulting apparent Young’s and shear modulus show strong dependence on the sphere packing density which is roughly linear. For high packing densities, however, the moduli increase slightly stronger.

### Comparison to experiments

To validate the accuracy of the computational models presented in Sect. 2, we set up the computations with the material parameters given in Table 1, the epoxy volume fraction of $16.2\%$ and the sphere packing density of $53.3\%$, as described in Sect.  2.2. By prescribing six linearly independent strain loadings, the components of the effective stiffness tensor (3.6) are determined through Hooke’s Law (3.4). The effective stiffness is assumed to be isotropic. This hypothesis is supported by the previously discussed findings [1] and the results of Sect. 3.4. Therefore, an isotropic approximation to the effective stiffness, defined via the shear and compression modulus, is calculated and subsequently compared to the results of experimental preinvestigations, see Sect.  2.2. The Young’s modulus was found to be 2.71 GPa, while the shear modulus was determined to be 1.12 GPa. The computations, on the other hand, delivered a Young’s modulus of 2.94 GPa and a shear modulus of 1.17 GPa. This corresponds to an error of approximately 8% for the Young’s modulus and an error of around 4% for the shear modulus. Furthermore, Zhou et al. [15] present the elastic properties of a different HSC specimen in their work. The production process mirrors the one described in Sect. 2.1. The resulting HSC was analyzed using the IET, see Sect.  2.2, which yielded a Young’s modulus of 1.865 GPa and a shear modulus of 0.793 GPa. Furthermore, Zhou et al. [15] present the sphere size distribution, but the exact volume fraction of the epoxy phase and the shell thickness of the HS were not identified. Nevertheless, the nominal thickness is given as 40 $\upmu$m, and we estimated the volume fraction of the epoxy phase to be roughly $10\%$, based on the given photographs of the microstructure. In addition, we assume that the sphere packing density is similar to the one determined for the HSC described in Sect. 2.1. Based on these assumptions, we computed a Young’s modulus of 1.8 GPa and a shear modulus of 0.76 GPa. This corresponds to a relative error of under $4\%$ percent for the Young’s modulus and just over $4\%$ for the shear modulus. In Table 3, the comparison of the experiments and simulations are presented.

**Table 3:** Comparison of experimentally and computationally determined isotropic moduli

IET (HSC: Sect. 2.1)	$E=2.71$ GPa,	$G=1.12$ GPa
computation	$E=2.94$ GPa,	$G=1.17$ GPa
IET (HSC: [15])	$E=1.865$ GPa,	$G=0.793$ GPa
computation	$E=1.80$ GPa,	$G=0.76$ GPa

## Conclusion

This work is concerned with modeling the microstructure and computing the effective elastic properties of glued metallic hollow-sphere composites used in sandwich materials for damping in structural parts of machine tools. Based on an initial sphere packing, introducing the glue between the spheres required significant work. We presented a novel method to represent the signed distance function of the glue-void interface exactly. This representation turned out to be critical for the subsequent applications because it was required for the composite-voxel method which ensured sufficient accuracy for the mesh sizes considered. Indeed, dispensing with composite voxels and working with a plain regular grid was shown to lead to significant errors even for fine resolutions. The introduced model enabled us to capture the isotropic material behavior of the HS without relying on regular sphere arrangements or restricting to small volume fractions of glue.

The presented modeling approach for HSCs comprehensively considers multiple sources of modeling error. For instance, it takes the volume fraction of epoxy via the bulk density of the spheres into account. The computed elastic properties of the HSC align well with the experimental results, confirming the isotropic material properties of the HSC. This agreement allows us to investigate how microstructure parameters influence the mechanical properties of HSCs. Our findings indicate that sphere shell thickness, epoxy volume fraction, and sphere packing density significantly impact the elastic properties of HSCs, whereas the sphere size distribution has minimal effect. Using the FFT-based homogenization methods enabled us to analyze the local fields and showed strong stress localization in the shells, hinting at possible failure mechanisms.

We conclude that the described modeling approach turns out to be reasonable, and it holds the potential for future work to extend this model to elucidate more characteristics of such composites. As a natural next step, the damping behavior should be considered, which is the core motivation for using HSCs in machine tool structures, and is inherently a nonlinear phenomenon. Resolving the underlying physics within the FFT-based framework adopted here is more involved than the linear elastic case and requires dedicated extensions for rate- and amplitude-dependent constitutive laws. The explicit level-set representation developed in this work provides a suitable starting point for such extensions. Further augmentations, for instance, toward predicting the yield strength of HSCs, are equally within reach.

## Data Availability

No datasets were generated or analyzed during the current study.

## Derivation of the alternative representation for the Dirichlet cells

The aim of this appendix is to derive the formulas necessary to define the Dirichlet tessellation and the equivalent representation as an intersection of half-spaces.

We start with a closer look at the definition of an arbitrary Dirichlet cell $\mathcal {D}_{b_i}$

A.1 $$\begin{aligned} \mathcal {D}_{b_i}&= \left\{ {\boldsymbol{ x}}\in \mathbb {R}^d~\left| ~\Pi _i({\boldsymbol{ x}})\le \Pi _j({\boldsymbol{ x}}),~\forall j\in \mathcal {I}_B\right. \right\} , \end{aligned}$$

Inserting the definition $\Pi _i({\boldsymbol{ x}}) = \left\| {\boldsymbol{ x}}-{\boldsymbol{ c}}_i\right\| ^2-r_i^2$ and rearranging gives us

A.2 $$\begin{aligned} \Pi _i({\boldsymbol{ x}})&\le \Pi _j({\boldsymbol{ x}}) \end{aligned}$$

A.3 $$\begin{aligned} \Leftrightarrow \quad \left\| {\boldsymbol{ x}}-{\boldsymbol{ c}}_i\right\| ^2-r_i^2&\le \left\| {\boldsymbol{ x}}-{\boldsymbol{ c}}_j\right\| ^2-r_j^2\end{aligned}$$

A.4 $$\begin{aligned} \Leftrightarrow \quad {\boldsymbol{ x}}^2 - 2{\boldsymbol{ x}}\cdot {\boldsymbol{ c}}_i + {\boldsymbol{ c}}_i^2 - r_i^2&\le {\boldsymbol{ x}}^2 - 2x\cdot {\boldsymbol{ c}}_j + {\boldsymbol{ c}}_j^2 - r_j^2\end{aligned}$$

A.5 $$\begin{aligned} \Leftrightarrow \quad - 2{\boldsymbol{ x}}\cdot \left( {\boldsymbol{ c}}_i - {\boldsymbol{ c}}_j\right)&\le -{\boldsymbol{ c}}_i^2 + {\boldsymbol{ c}}_j^2 - r_j^2 + r_i^2\end{aligned}$$

A.6 $$\begin{aligned} \Leftrightarrow \quad \underbrace{\left( {\boldsymbol{ c}}_i - {\boldsymbol{ c}}_j\right) }_{=:{\boldsymbol{ n}}_{ij} \left\| {\boldsymbol{ c}}_i-{\boldsymbol{ c}}_j\right\| }{\boldsymbol{ x}}&\ge \frac{{\boldsymbol{ c}}_i^2 - {\boldsymbol{ c}}_j^2 + r_j^2 - r_i^2}{2}\end{aligned}$$

A.7 $$\begin{aligned} \Leftrightarrow \quad {\boldsymbol{ n}}_{ij}\cdot {\boldsymbol{ x}}&\ge \frac{{\boldsymbol{ c}}_i^2 - {\boldsymbol{ c}}_j^2 + r_j^2 - r_i^2}{2\left\| {\boldsymbol{ c}}_i-{\boldsymbol{ c}}_j\right\| } =: d_{ij}, \end{aligned}$$

where the condition retrieved in (A.7) characterizes a half-space. Inserting Eq. (A.7) into the original definition of the Dirichlet cells (A.1) subsequently gives rise to the interpretation of a Dirichlet cell as the intersection of half-spaces, i.e., a convex polytope:

A.8 $$\begin{aligned} \mathcal {D}_{b_i}&= \left\{ {\boldsymbol{ x}}\in \mathbb {R}^d~\left| ~\Pi _i({\boldsymbol{ x}})\le \Pi _j({\boldsymbol{ x}}),~\forall j\in \mathcal {I}_B\right. \right\} \end{aligned}$$

A.9 $$\begin{aligned}&= \left\{ {\boldsymbol{ x}}\in \mathbb {R}^d~\left| ~{\boldsymbol{ n}}_{ij}\cdot {\boldsymbol{ x}}\ge d_{ij}, ~\forall j\in \mathcal {I}_B\right. \right\} \end{aligned}$$

A.10 $$\begin{aligned}&= \underset{j\in \mathcal {I}_B}{\bigcap }h_{ij} \end{aligned}$$

with $$h_{ij} = \left\{ {\boldsymbol{ x}}\in \mathbb {R}^d~\left| ~{\boldsymbol{ n}}_{ij}\cdot {\boldsymbol{ x}}\ge d_{ij}\right. \right\} $$.

Even though the definition presented in Eq. (A.10) theoretically suffices for all uses within this paper, it still considers all possible indices $j\in \mathcal {I}_B$. To decrease the effort in handling a Dirichlet cell it is advantageous to drop the redundant half-spaces. We call a half-space *h* redundant if removing it does not change the resulting polytope, otherwise it is called active. A possible criterion for the redundancy of a half-space can be found by inspecting the vertex representation

A.11 $$\begin{aligned} \mathcal {D}_{b_i} = \left\{ {\boldsymbol{ x}}\in \mathbb {R}^{d}~\left| ~{\boldsymbol{ x}}={\boldsymbol{ v}}_k + t ({\boldsymbol{ v}}_k-{\boldsymbol{ v}}_l),~t\in \left[ 0,1\right] ,~\forall {\boldsymbol{ v}}_k,{\boldsymbol{ v}}_l\in \mathcal {V}_{\mathcal {D}}\right. \right\} \end{aligned}$$

of a convex polytope, with $\mathcal {V}_{\mathcal {D}}$ denoting the vertex set of the Dirichlet cell. In the context of a half-space *h*, where *n* and *d* denote the characteristics of the plane that define it, it is considered redundant if none of the vertices $v\in \mathcal {V}_{\mathcal {D}}$ satisfy the equation ${\boldsymbol{ n}}\cdot {\boldsymbol{ v}}=d$. This finally leads us to a description of the Dirichlet cell only using active half-spaces

A.12 $$\begin{aligned} \mathcal {D}_{b_i}&= \underset{j\in \mathcal {I}_{\mathcal {H}_i}}{\bigcap }h_{ij},\quad \text {with}\quad \mathcal {I}_{\mathcal {H}_i} = \left\{ j\in \mathcal {I}_B~\left| ~\exists {\boldsymbol{ v}}\in \mathcal {V}_{\mathcal {D}}, {\boldsymbol{ v}}\in h_{ij}\right. \right\} . \end{aligned}$$

## The level set function of a union of d-balls

The aim of this appendix is to introduce the level set function for the union of *d*-dimensional balls *B*.

To develop an explicit formula for the distance to the surface $\partial B$, some preliminary definitions are in order. For each ball, we determine the corresponding the Dirichlet cell $\mathcal {D}_{b_i}$, in accordance with Apx. A. In particular, we determine the set of active half-spaces $\mathcal {H}_i$. Each half-space $h\in \mathcal {H}_i$ is defined via a hyperplane with a normal direction ${\boldsymbol{ n}}$ and the distance from the origin *d* using the Hesse normal form. For the level set function, we need to determine the distance to the surface of the union of balls. The surface of the union of balls $\partial B$ can be represented as the union of the intersections of the Dirichlet cells with the surfaces of their corresponding balls [49], i.e., we decompose the surface of the union of balls $\partial B$ via

B.1 $$\begin{aligned} \partial B = \text {ext}(B)= \underset{i\in \mathcal {I}_B}{\bigcup }\partial \overline{b}_i \quad \text {with}\quad \partial \overline{b}_i = \partial b_i\cap \mathcal {D}_{b_i}. \end{aligned}$$

Next, we define the point

B.2 $$\begin{aligned} {\boldsymbol{ x}}^*= \underset{\overline{{\boldsymbol{ x}}}\in \partial B}{\text {argmin}}\left\| {\boldsymbol{ x}}-\overline{{\boldsymbol{ x}}}\right\| , \end{aligned}$$

representing the point on the surface $\partial B$ closest to ${\boldsymbol{ x}}\in \mathcal {D}_{b_i}$. To determine the (or a) point ${\boldsymbol{ x}}^*$, we introduce a series of projections on the surface of the intersection $\mathcal {D}_{b_i}\cap b_i$. The surface of the Dirichlet cell $\mathcal {D}_{b_i}$ is described via hyperplanes. We select all possible combinations of $k\in \mathbb {N}$ half-spaces, with $k\le d$, in the sets

B.3 $$\begin{aligned} \mathcal {S}^{(k)}_{i} = \left\{ S \subseteq \mathcal {H}_i ~\left| ~ |S| = k \right. \right\} . \end{aligned}$$

The parts of the surface of the Dirichlet cell $\mathcal {D}_{bi}$ which intersect with the ball $b_i$ are of particular interest. Therefore, we form the intersections of hyperplanes describing the half-spaces contained in the sets $S\in \mathcal {S}^{(k)}_{i}$ with the ball $b_i$. The intersection of *k* hyperplanes and a *d*-ball gives rise to a $(d-k)$-ball or an empty set [49]. We collect all non-empty intersections in the sets:

B.4 $$\begin{aligned} \mathcal {B}^{(k)}_i&= \left\{ b\subset \mathbb {R}^d ~\left| ~ \exists S\in \mathcal {S}^{(k)}_{i}:~b = \bigcap _{h \in S} \left( \text {ext}(h)\cap b_i\right) \text { and } b\ne \emptyset \right. \right\} \text { with } k>0, \end{aligned}$$

where we define $\mathcal {B}^{(0)}_i = \left\{ b_i\right\}$. Each $(d-k)$-ball $b\in \mathcal {B}^{(k)}_i$ is furnished with the radial projection

B.5 $$\begin{aligned} \overline{P}_b({\boldsymbol{ x}})&= \frac{{\boldsymbol{ x}}-{\boldsymbol{ c}}}{\left\| {\boldsymbol{ x}}-{\boldsymbol{ c}}\right\| }r+{\boldsymbol{ c}} \end{aligned}$$

of an arbitrary point ${\boldsymbol{ x}}\in b$ onto the surface $\partial b$ of the ball *b*, where ${\boldsymbol{ c}}$ denotes the center and *r* the radius of the ball *b*. Next, we define the projection of the arbitrary point ${\boldsymbol{ x}}\in b$ onto a half-space $h\in \mathcal {H}_i$:

B.6 $$\begin{aligned} \tilde{P}_{b,h}({\boldsymbol{ x}}) = {\boldsymbol{ x}}-{\boldsymbol{ n}}\left( {\boldsymbol{ n}}\cdot {\boldsymbol{ x}}- d\right) . \end{aligned}$$

To project the point ${\boldsymbol{ x}}$ onto the surface of the Dirichlet-cell $\mathcal {D}_{b_i}$, we select the hyperplane $\tilde{h}\in \mathcal {H}_i$ for which minimizes the distance between the original point and the projected point. We determine the hyperplane $\tilde{h}$ via

B.7 $$\begin{aligned} \tilde{h} = \underset{h\in \mathcal {H}}{\text {argmin}}\left\| {\boldsymbol{ x}}-\tilde{P}_{b,h}({\boldsymbol{ x}})\right\| . \end{aligned}$$

Last but not least, we define a projection for each ball, which represents a selection from one of the two projections (B.5) or (B.6), s.t. we either project a candidate point onto the surface of the ball *b* or onto the surface of the Dirichlet cell:

B.8 $$\begin{aligned} P_b({\boldsymbol{ x}})&=\left\{ \begin{array}{rl} \overline{P}_b({\boldsymbol{ x}}), &  \overline{P}_b({\boldsymbol{ x}})\in b,\\ \tilde{P}_{b,\tilde{h}}({\boldsymbol{ x}}), & \text {otherwise}. \end{array} \right. \end{aligned}$$

For the projection (B.8), we observe two cases:

1. $\overline{P}_b({\boldsymbol{ x}})\in b$ holds, i.e., the projection $P_b$ maps the point ${\boldsymbol{ x}}$ onto the surface of the ball *b* and subsequently onto the surface of the ball $b_i$. Put differently, we found a point ${\boldsymbol{ x}}^{*}$ on the surface $\partial B$ with the smallest distance to the point ${\boldsymbol{ x}}$.
2. If $\overline{P}_b({\boldsymbol{ x}})\notin b$ holds, we obtain the projection of the point ${\boldsymbol{ x}}$ onto the hyperplane $\tilde{h}$. Subsequently, the projected point $\tilde{P}_{b,\tilde{h}}({\boldsymbol{ x}})$ is an element of a ball, which results from the intersection of the ball *b* with the hyperplane of $\tilde{h}$, we refer to the resulting ball of this intersection as $\tilde{b}$. As we assume the ball *b* to be an element of the set $\mathcal {B}^{(k)}_i$ it follows that the ball $\tilde{b}$ is an element of the set $\mathcal {B}^{(k+1)}_i$.

The second case motivates to repeat the projection for the lower dimensional ball $\tilde{b}$, that the point ${\boldsymbol{ x}}$ is projected onto via the projection $\tilde{P}_{b,\tilde{h}}$, in the hope that the point resulting from this projection is part of the surface of the union of balls $\partial B$. We therefore obtain the projection $P_{\tilde{b}}(\tilde{P}_{b,\tilde{h}}({\boldsymbol{ x}}))$, where the same two aforementioned cases can occur. Repeating the arguments, we are led to a series of $(d-k)$-projections, eventually leading to a point that is either contained in the surface of the ball $b_i$ or lies on a ball of the set $\mathcal {B}^{(d)}_i$ within the ball $b_i$. Thus far, we considered an arbitrary ball *b*. By choosing $b_i$ as the starting point for the projection series, we obtain a series of *d* projections for an arbitrary point ${\boldsymbol{ x}}$ which is expressed by

B.9 $$\begin{aligned} P_i({\boldsymbol{ x}}) = P_{b^{(1)}_{i}({\boldsymbol{ x}})}\left( P_{b^{(2)}_{i}({\boldsymbol{ x}})}\left( \dots P_{b^{(d-1)}_{i}({\boldsymbol{ x}})}\left( P_{b^{(d)}_{i}({\boldsymbol{ x}})}\left( {\boldsymbol{ x}}\right) \right) \right) \right) . \end{aligned}$$

Furthermore, we introduce the series of balls $b^{(k)}_{i}({\boldsymbol{ x}})$, for $k=1,\dots ,d$, representing the sequence of balls onto which we project the individual points. Each sequence starts with $b^{(d)}_{i}({\boldsymbol{ x}}) = b_i$. We wish to stress that the subsequent projections do explicitly depend on the initial point ${\boldsymbol{ x}}$. This dependency stems from the half-spaces or ball surfaces we projected the point ${\boldsymbol{ x}}$ onto, see Eq. (B.7). The resulting sequence is given by

B.10 $$\begin{aligned} b^{(k)}_{i}({\boldsymbol{ x}}) = \left\{ \begin{array}{rl} \text {ext}(\tilde{h})\cap b^{(k+1)}_{i}({\boldsymbol{ x}}) , &  P_{b^{(k+1)}_{i}({\boldsymbol{ x}})}\notin \partial B,\\ e_c\cap b^{(k+1)}_{i}({\boldsymbol{ x}}) , & \text {otherwise}, \end{array} \right. \end{aligned}$$

where $e_c$ represents a hyperplane containing the point ${\boldsymbol{ x}}$ and the center of the ball $b_i$. Consequently, all subsequent projections turn out to be the same point. In the first case, we obtain the previously discussed projection onto a lower dimensional ball.

The described procedure finally enables us to retrieve a point ${\boldsymbol{ x}}^*$

B.11 $$\begin{aligned} {\boldsymbol{ x}}^*&= \left\{ \begin{array}{rl} P_i({\boldsymbol{ x}}), &  P_i({\boldsymbol{ x}})\in \partial b_i,\\ \underset{\overline{{\boldsymbol{ x}}}\in \mathcal {V}}{\text {argmin}}\left\| {\boldsymbol{ x}}-\overline{{\boldsymbol{ x}}}\right\| , & \text {otherwise}, \end{array} \right. \end{aligned}$$

B.12 $$\begin{aligned} \text {with } \mathcal {V}&= \left\{ {\boldsymbol{ x}}\in \mathbb {R}^d~\left| ~ {\boldsymbol{ x}}\in \partial B,~\exists i \in \mathcal {I}_B,~\exists b\in \mathcal {B}^{(d)}_i:~{\boldsymbol{ x}}\in b \right. \right\} . \end{aligned}$$

To determine the point ${\boldsymbol{ x}}^*$, see Eq. (B.11), we distinguish two cases:

1. The projection of the point ${\boldsymbol{ x}}$ lies on the surface of the ball $b_i$. This point then minimizes the right-hand side of the condition (B.2) and therefore represents the point ${\boldsymbol{ x}}^*$.
2. The projected point $P_{b_i}({\boldsymbol{ x}})$ does not lie on the surface $\partial b_i$, i.e., the point $P_{b_i}({\boldsymbol{ x}})$ lies on a supporting plane of the Dirichlet cell. In this case, the minimum distance to the surface is realized for a point contained on the vertex set $\mathcal {V}$.

Eventually, we are led to the desired description

B.13 $$\begin{aligned} \Gamma _i({\boldsymbol{ x}}) = \left\{ \begin{array}{rl} -\left\| {\boldsymbol{ x}}-{\boldsymbol{ x}}^*\right\| , & {\boldsymbol{ x}}\in B,\\ \underset{i\in \mathcal {I}_B}{\text {min}} \left\| {\boldsymbol{ x}}-{\boldsymbol{ c}}_i\right\| -r_i, & \text {otherwise}. \end{array} \right. \end{aligned}$$

The three-dimensional case was motivated in Sect. 2.4.
